# Dodecyl creatine ester improves cognitive function and identifies key protein drivers including KIF1A and PLCB1 in a mouse model of creatine transporter deficiency

**DOI:** 10.3389/fnmol.2023.1118707

**Published:** 2023-03-24

**Authors:** Aloïse Mabondzo, Rania Harati, Léa Broca-Brisson, Anne-Cécile Guyot, Narciso Costa, Francesco Cacciante, Elena Putignano, Laura Baroncelli, Matthew R. Skelton, Cathy Saab, Emmanuelle Martini, Henri Benech, Thomas Joudinaud, Jean-Charles Gaillard, Jean Armengaud, Rifat Hamoudi

**Affiliations:** ^1^Université Paris Saclay, CEA, INRAE, Département Médicaments et Technologies pour la Santé (MTS), Gif sur Yvette, France; ^2^Department of Pharmacy Practice and Pharmacotherapeutics, College of Pharmacy, University of Sharja, Sharjah, United Arab Emirates; ^3^Sharjah Institute for Medical Research, University of Sharjah, Sharjah, United Arab Emirates; ^4^Institute of Neuroscience, National Research Council (CNR), Pisa, Italy; ^5^Department of Developmental Neuroscience, IRCCS Stella Maris Foundation, Pisa, Italy; ^6^Department of Pediatrics, University of Cincinnati College of Medicine and Division of Neurology, Cincinnati Children’s Research Foundation, Cincinnati, OH, United States; ^7^Université de Paris and Université Paris Saclay, CEA, Stabilité Génétique Cellules Souches et Radiations, Fontenay aux Roses, France; ^8^Ceres Brain Therapeutics, Paris, France; ^9^Université Paris Saclay, CEA, Département Médicaments et Technologies pour la Santé (MTS), INRAE, Bagnol sur Cèze, France; ^10^Clinical Sciences Department, College of Medicine, University of Sharjah, Sharjah, United Arab Emirates; ^11^Division of Surgery and Interventional Science, University College London, London, United Kingdom

**Keywords:** dodecyl creatine ester, creatine transporter deficiency, target identification, cognitive function, CTD-pathophysiology

## Abstract

Creatine transporter deficiency (CTD), a leading cause of intellectual disability is a result of the mutation in the gene encoding the creatine transporter SLC6A8, which prevents creatine uptake into the brain, causing mental retardation, expressive speech and language delay, autistic-like behavior and epilepsy. Preclinical *in vitro* and *in vivo* data indicate that dodecyl creatine ester (DCE) which increases the creatine brain content, might be a therapeutic option for CTD patients. To gain a better understanding of the pathophysiology and DCE treatment efficacy in CTD, this study focuses on the identification of biomarkers related to cognitive improvement in a Slc6a8 knockout mouse model (Slc6a8−/y) engineered to mimic the clinical features of CTD patients which have low brain creatine content. Shotgun proteomics analysis of 4,035 proteins in four different brain regions; the cerebellum, cortex, hippocampus (associated with cognitive functions) and brain stem, and muscle as a control, was performed in 24 mice. Comparison of the protein abundance in the four brain regions between DCE-treated intranasally Slc6a8−/y mice and wild type and DCE-treated Slc6a8−/y and vehicle group identified 14 biomarkers, shedding light on the mechanism of action of DCE. Integrative bioinformatics and statistical modeling identified key proteins in CTD, including KIF1A and PLCB1. The abundance of these proteins in the four brain regions was significantly correlated with both the object recognition and the Y-maze tests. Our findings suggest a major role for PLCB1, KIF1A, and associated molecules in the pathogenesis of CTD.

## Introduction

Neurodevelopmental disorders represent a significant health problem due to the heterogeneity of the underlying causes and a lack of appropriate treatment options. Creatine transporter deficiency (CTD) is a rare genetic disorder and a subset of intellectual disability (ID). However its symptoms including autism-like symptoms with ID, expressive speech and language delay, movement disorders, and epilepsy (Salomons et al., 2001; Stockler et al., 2007; Des Roches et al., 2015) overlaps with those of common neurodevelopmental disorders. CTD is an X-linked (XL) disorder caused by mutations in *SLC6A8,* the gene encoding creatine transporter (CrT)(Braissant et al., 2011), that prevent the transport of creatine (Cr), which is essential for brain function, into the brain (van de Kamp et al., 2014) and is estimated to be the cause of 1%–2% of all cases of XL ID (Cheillan et al., 2012; Des Roches et al., 2015) and about 1% of cases with ID of unknown aethiology (Clark et al., 2006). As expected, the symptoms are most severe in males, with female carriers presenting with a milder phenotype. Some of the main difficulties in elucidating the pathogenesis of and treating ID are that there are wide variety of causes of ID, with no single cause being associated with a significant majority of ID cases.

Several combinations of nutritional supplements or Cr precursors l-arginine and l-glycine, have been studied as therapeutic approaches for CTD, but they have shown very limited success (Valayannopoulos et al., 2013; Jaggumantri et al., 2015; Bruun et al., 2018). However, our previous findings suggest that following the inhalation of dodecyl creatine ester (DCE) in Slc6a8^−/y^ (CrT KO) mice, an animal model recapitulating the clinical features of human CTD, an increase of Cr brain content and synaptic markers could be achieved in the synapsis terminals and thus improving the cognitive function of Slc6a8−/y mice. These findings highlight that DCE might be a therapeutic option for CTD patients (Trotier-Faurion et al., 2013, 2015; Ullio-Gamboa et al., 2019).

To gain a better understanding of the pathophysiology of CTD and DCE treatment efficacy, we focused in this study on the identification of biomarkers related to cognitive improvement in a Slc6a8−/y mice which have low Cr content in the brain and hippocampus (Baroncelli et al., 2016). This mouse model exhibits a precocious cognitive and autistic-like defects, mimicking the early key features of human congenital creatine deficiency syndromes. Moreover, mutant mice displayed a progressive impairment of short and long-term declarative memory denoting an early brain aging. To that end, the study employed a combination of cognitive tests and molecular methods to decipher some of the molecular mechanisms involved in CTD pathophysiology. The cognitive tests included the use of object recognition test (ORT), Y-maze and Morris water maze (MWM) tests to show the decline of cognitive function in *Slc6a8^−/y^* mice and whether the treatment with DCE improves the cognitive function. The molecular methods involved the application of shotgun proteomics to four different brain regions; the cerebellum, cortex, hippocampus (associated with cognitive functions) and brain stem, and muscle as a control.

## Results

### Restoration of cognitive functions in DCE-treated creatine knockout mice

DCE was intranasally administered as previously reported (Ullio-Gamboa et al., 2019) to CrT KO mice for 30 days, while wild-type (WT) and vehicle-treated mice were used as controls (*N* = 8 per group). A volume of 6 μL of DCE or vehicle was placed in the nostril. DCE (4 mg/g) or vehicle was given twice bilaterally (12 μL total volume). Consistent with previous findings, CrT KO mice showed object recognition deficits when compared with WT mice. Mice were assigned to treatment groups by sorting animals based on discrimination index (DI) and alternating assignments between vehicle and treatment to avoid performance confounds.

**Figure 1:**
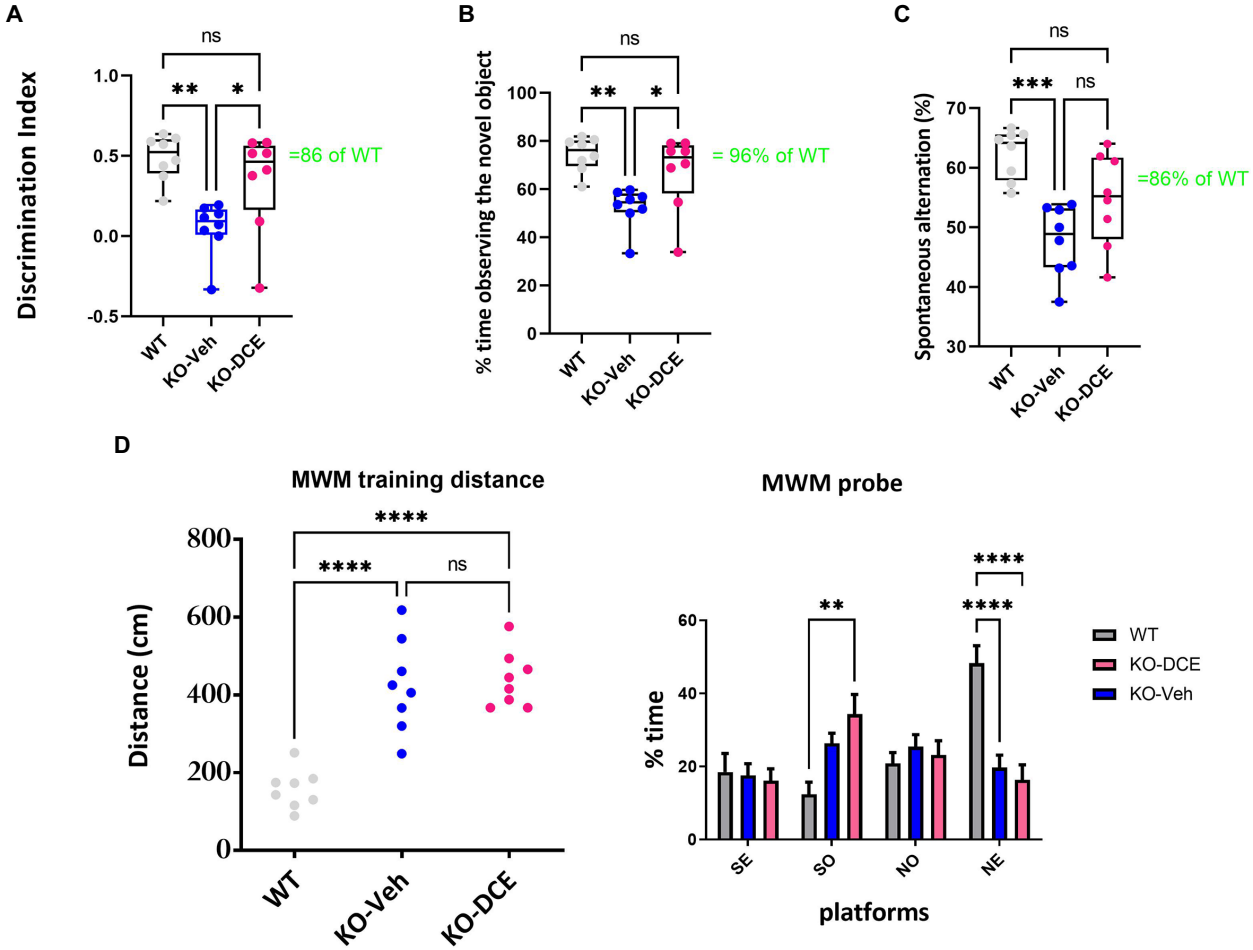
Analysis of cognition among the different experimental groups. **(A,B)** Object recognition in the ORT was impaired in CrT KO mice. **(A)**, Analysis of the object discrimination index and **(B)**, percentage of time spent exploring the novel object revealed that WT and DCE-treated CrT KO mice but not vehicle-treated CrT KO mice showed a preference for the novel object. **(C)** Early deficiency of working and spatial memory in CrT KO mice measured by the Y-maze test. CrT KO mice showed a change in the spontaneous alternation percentage in the Y-maze test, which was significantly improved by DCE treatment. **(D)** CrT deletion progressively deteriorates spatial learning and memory in KO mice. Left, learning plot for the three group of animals (WT, CrT KO mice and DCE-treated KO mice). A significant difference was detected between WT and CrT KO mice as well as between WT and DCE-treatd CrT KO mice (*p* < 0.0001). Right histograms showing the mean swimming path covered to locate the submerged platform on the last day of training. Four starting positions arbitrarily designated North (N), South (S), East (E), and West (W) were selected, thus dividing into 4 quadarants were the mice were allowed to search for the escape platform. A significant difference was detected between WT and DCE-treated CrT KO mice in the SO position (*p* < 0.001). In the NE position, a significant difference was detected between WT and DCE-treated KO-DCE mice as well as between WT and CrT KO mice (*p* < 0.0001). The data are the mean ± s.e.m. Statistical analysis was performed by one-way or two way ANOVA followed by Tukey’s post-hoc test or 2 way ANVOA followed by. *p < 0.05; **p < 0.001;***p < 0.0001; ns = not significant.

Figure 1A shows DI data. Vehicle-treated CrT KO mice showed reductions in DI compared with both WT mice (*p* = 0.023) and DCE-treated CrT KO mice (*p* < 0.05). DCE-treated CrT KO mice spent more time exploring the novel object than vehicle-treated CrT KO mice (one-way ANOVA, *p* < 0.01; Tukey’s *post hoc* test, p < 0.05; Figure 1B), but there was no difference between DCE-treated CrT KO mice and WT mice (*p* = 0.406). Strikingly, the median exploration time for the DCE-treated CrT KO mice was 96% of the median exploration time for the WT mice.

We used the Y-maze test measuring the spontaneous alternation rate, the single parameter with the highest accuracy in discriminating WT and CrT KO mice (Mazziotti et al., 2020). A reduction of the spontaneous alternation rate in vehicle-treated CrT KO mice (48%) was observed compared to WT mice (62%; one-way ANOVA, *p* < 0.001; Tukey’s *post hoc* test, p < 0.001; Figure 1C). The median spontaneous alternation rate was higher in DCE-treated CrT KO mice than in vehicle-treated CrT KO mice but did not differ between DCE-treated CrT KO mice and WT mice (86%; *p* = 0.058; Figure 1C).

Memory was assessed using the MWM test. The results show that DCE did not have a beneficial effect on the performance of CrT KO mice in the MWM test (Figure 1D), suggesting that DCE treatment ameliorates some cognitive deficits seen in these mice.

### Proteomic analysis identified unique proteins abundant in specific brain regions

In order to identify proteins across the different brain regions involved in the pathogenesis of CTD, proteomics based differential abundance analysis was performed in the different brain regions of 24 mice. Immediately after behavioral testing, the animals from all three groups were sacrificed. Label-free shotgun proteomics analysis of cortical, hippocampal, cerebellar, and brainstem tissues and muscle tissue as a control was carried out for each mouse in WT, vehicle-treated CrT KO mice and DCE-treated CrT KO mice.

High-resolution tandem mass spectrometry (MS) analysis of the 120 biological samples generated a large dataset comprising of 7,006,153 MS/MS spectra showing the abundance of 4,035 proteins for the five tissues from each mouse. Following unsupervised filtering and normalization (Hachim et al., 2020) (Supplementary Table 1), the proteins whose abundance was significantly altered by mutant CrT and DCE treatment were identified using reproducibility-optimized statistical testing in each of the five tissue types. The workflow of the bioinformatics analysis is described in Supplementary Figure 1.

The muscle was considered a control for protein analysis due to the fact that Cr levels are decreased in muscle as well. Even if the mice do have a muscle phenotype, we have shown that the learning deficits are independent of any somatic problems the mice may have hence in this study we wanted to compare various regions of the brain to tissue that is not related to the brain, which in this case is the muscle. In addition, we did a global coefficient of variation (CV) calculation of all the proteins abundance between DCE, Veh, and WT for each of the 4 brain regions as well as muscle. The results show that the least variation is in the muscle tissue with CV less than 4,000. However, all the 4 brain regions had CV of more than 5,000 [cortex (5692.59), hippocampus (5166.71), brain stem (5519.19)] with cerebellum being the highest at 5948. This shows that there are some alteration in muscle but not as much as we see in the 4 different brain regions.

**Figure 2:**
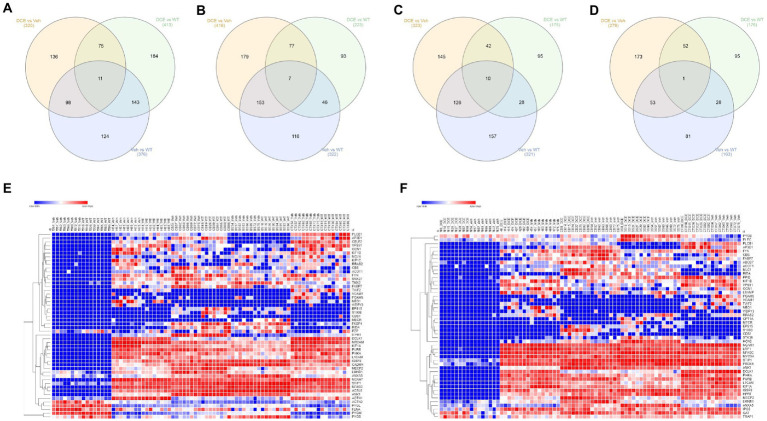
Comparison of proteomic signatures in different brain regions among the different experimental groups**. (A–D)** Venn diagram showing overlapping proteins among the three experimental groups. The proteins that showed a significant change in abundance in CrT KO mice compared with the WT and in DCE-treated mice compared with vehicle-treated mice were selected for pathway analysis. The proteins that showed a change in abundance in the vehicle-treated mice and the DCE-treated mice compared with the WT mice were also selected for pathway analysis. The abundances of these proteins in the cortex, cerebellum, brainstem, hippocampus and muscle were analyzed using a multivariate statistical model based on one-way ANOVA followed by Bonferroni’s *post hoc* test. **(E)** Heatmap showing the proteins that showed a significant change in abundance in vehicle-treated CrT KO mice compared WT mice. **(F)** Heatmap showing the proteins that showed a significant change in abundance in DCE-treated CrT KO mice compared with vehicle-treated CrT KO mice.

A total of 376, 322, 163, and 321 proteins were found to be differentially abundant in the cortex, cerebellum, brainstem and hippocampus, respectively, between WT mice and vehicle-treated CrT KO mice. The abundances of 320, 416, 279, and 323 proteins in the cortex, cerebellum, brainstem and hippocampus, respectively, were marked altered in DCE-treated CrT KO mice compared with vehicle-treated CrT KO mice (Supplementary Figures 2A–D, 3B,D,F,H), while 413, 223, 176 and 175 proteins were differentially abundant in DCE-treated CrT KO mice compared with WT mice (Supplementary Figures 3A,C,E,G). Overlapping proteins were observed in the comparisons of DCE-treated vs. vehicle-treated mice CrT KO, DCE-treated CrT KO vs. WT mice, and vehicle-treated CrT KO vs. WT mice. Most of the overlapping proteins in the comparison between DCE-treated vs. vehicle-treated mice CrT KO and vehicle-treated CrT KO vs. WT mice were found in the cortex (98 proteins) (Figure 2A), cerebellum (153 proteins) (Figure 2B), and hippocampus (126 proteins) (Figure 2C); conversely, fewer overlapping proteins were found in the brainstem (53 proteins) (Figure 2D; Supplementary Table 2). The proteins whose abundances were altered in vehicle-treated mice and DCE-treated mice compared with WT mice were also selected in cortex (11 proteins) (Figure 2A), cerebellum (7 proteins) (Figure 2B), hippocampus (10 proteins) (Figure 2C) and brain stem (1 protein) (Figure 2D; Supplementary Table 2).

The overlapping proteins whose abundances were significantly altered in all CrT KO mice compared to that in WT mice and in DCE-treated mice CrT KO mice compared to that in vehicle-treated CrT KO mice were then selected for pathway analysis using gene set enrichment analysis carried out using ENRICHR. The proteins found to be involved in different diseases and pathways were selected for subsequent analysis [Cortex (41 proteins), Hippocampus (53 proteins), Cerebellum (52 proteins), and brain stem (25 proteins)]. These findings suggest that lack of Cr into the brain of CrT KO mice leads to a significant alteration of protein abundance involved in the pathogenesis of CTD.

### Identification of a panel of 14 proteins in the brain involved in cognitive activity using a multivariate statistical model

**Figure 3:**
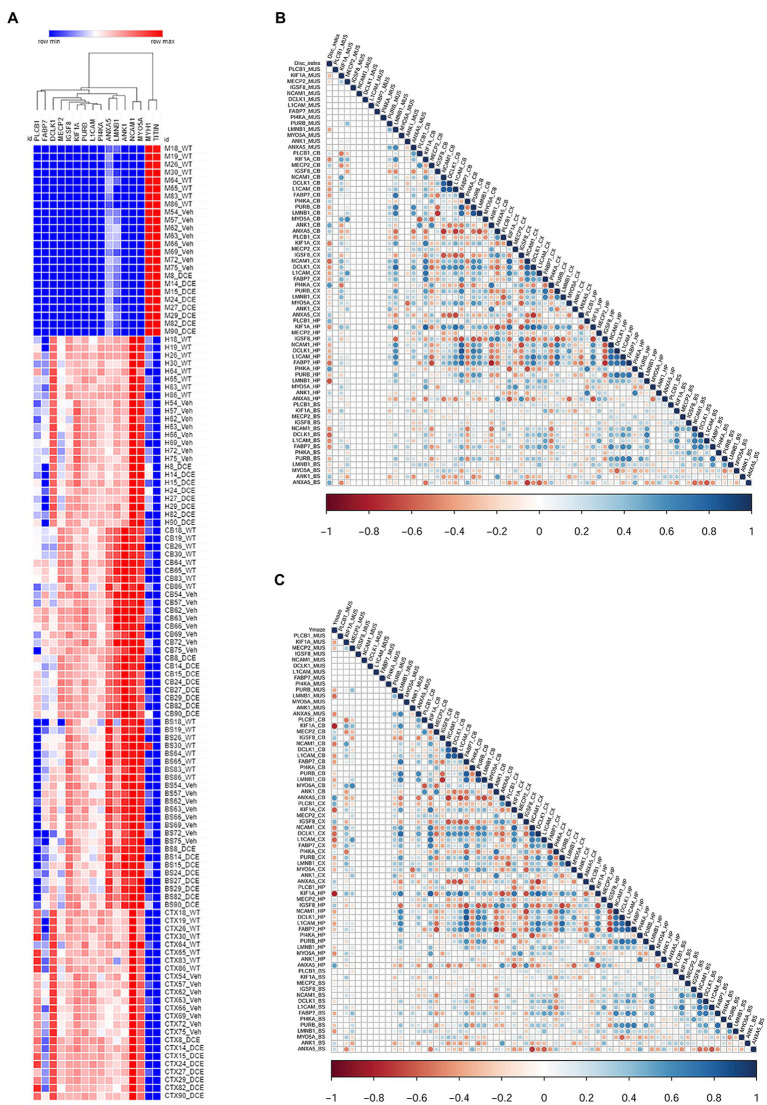
Correlation of the levels of 14 proteins with performance in different cognitive tests using a stepwise regression statistical model. **(A)** Heatmap showing the abundance levels of the 14 proteins that showed significant changes in abundance in both vehicle-treated CrT KO mice and DCE-treated CrT KO mice**. (B)** Map showing the correlation between the level of each of the 14 proteins with performance in the ORT to evaluate cognition. **(C)** Maps showing the correlation between the level of each of the 14 proteins and performance in the Y-maze test. The correlation maps were derived using a stepwise regression model to assess the correlation between the abundance each of the 14 differentially expressed proteins and performance in each of the cognitive tests. Group comparisons were carried out using a statistical model based on one-way ANOVA followed by Bonferroni’s *post hoc* test for multiple comparisons.

A multivariate statistical model comparing WT mice with vehicle-treated CrT KO mice (Figure 2E) and DCE-treated CrT KO mice with vehicle-treated CrT KO mice was used to identify key proteins (Figure 2F). Among the proteins whose abundances were affected by either CrT deficiency or DCE treatment, that were discussed in the previous section, bioinformatics analysis and statistical modelling identified 14 proteins that were the most abundant in the four different brain regions in both vehicle-treated and DCE-treated CrT KO mice. Those most abundant proteins were significantly altered by the mutation compared to WT(vehicle-treated vs. WT) and by the treatment compared to vehicle (DCE-treated vs. vehicle treated) and their abundance after treatment was restored to levels comparable to those in WT mice (Figure 3A; Supplementary Tables 3, 4).

The results showed that PLCB1 abundance in the cortex (*p* = 0.0003) (Supplementary Table 3a); MeCP2 (*p* = 0.022), ANK1 (*p* = 0.0001), and ANXA5 (*p* = 0.00004) abundance in the cerebellum (Supplementary Table 3c); and IGSF8 (*p* = 0.00036) abundance in the hippocampus (Supplementary Table 3b) were significantly downregulated in vehicle-treated CrT KO mice compared with WT mice, DCE treatment significantly rescued the abundance of these proteins in these brain regions in CrT KO mice (Figure 3A; Supplementary Table 4). In contrast, NCAM1 (*p* = 1.38E-07), PI4KA (*p* = 0.026), DCLK1 (*p* = 3^−15^) and PURB (*p* = 0.045) abundance in the cortex (Supplementary Table 3a); KIF1A (*p* = 2^−24^), NCAM1 (*p* = 0.038) and L1CAM (*p* = 0.001) abundance in the hippocampus (Supplementary Table 3b); and LMNB1 (*p* = 1E^−05^), FABP7 (*p* = 8.30E^−05^), and PURB (*p* = 0.03) abundance in the cerebellum (Supplemental Table 3c) were significantly upregulated in CrT-treated vehicle KO mice compared with WT mice but were not different between WT and DCE-treated CrT KO mice (Figure 3A; Supplementary Table 4).

### Correlation of the proteins with cognitive behavioral tests using stepwise regression statistical model shows KIF1A is abundant across the brain regions

A stepwise regression model was used to assess the effect of each of the proteins of interest on cognitive outcomes to identify those that may influence cognitive function. The levels of several proteins were significantly correlated with the discrimination index (DI); the correlation between KIF1A, Fabp7 and L1CAM levels and DI was found in the hippocampus, cortex, cerebellum and brain stem (*n* = 11, Supplementary Table 5a).

We also observed that the level of LMNB1, which is involved in cerebellar ataxia and adult autosomal dominant leukodystrophy (*r*^2^ = −0.634, *p* < 0.0001), and the level of Pi4KA (*r*^2^ = −0.634, *p* = 0.066) in the hippocampus were correlated with the DI. Several proteins were also significantly correlated with the performance in the Y-maze test (*n* = 11, Figure 3C, Supplementary Table 5b). The level of KIF1A, a kinesin that transports synaptic vesicle precursors, was most strongly correlated with the spontaneous alternation rate in the Y-maze test (hippocampus: *r*^2^ = −0.795, *p* < 0.0001; cortex: *r*^2^ = −0.608, *p* = 0.001; cerebellum: *r*^2^ = 0.774, *p* < 0.0001). Fabp7 and L1CAM correlated with the spontaneous alternation rate in the Y-maze test in the 3 brain regions (Supplementary Table 5b). In addition, PLCB1 is significantly more abundant in the cortex compared to other brain regions and its abundance in the cortex and hippocampus is correlated with DI and Y-maze (*p* = 0.01).

Amongst the 14 key proteins with individual animal performance in different cognitive tests, KIF1A was shown to be the protein with the most uniformly abundance across the 4 brain regions and correlated with the DI in the novel object recognition test and spontaneous alternation in the Y-maze test.

### KIF1A and PLCB1 interplay is associated with DCE treatment efficacy in CrT KO mice

**Figure 4:**
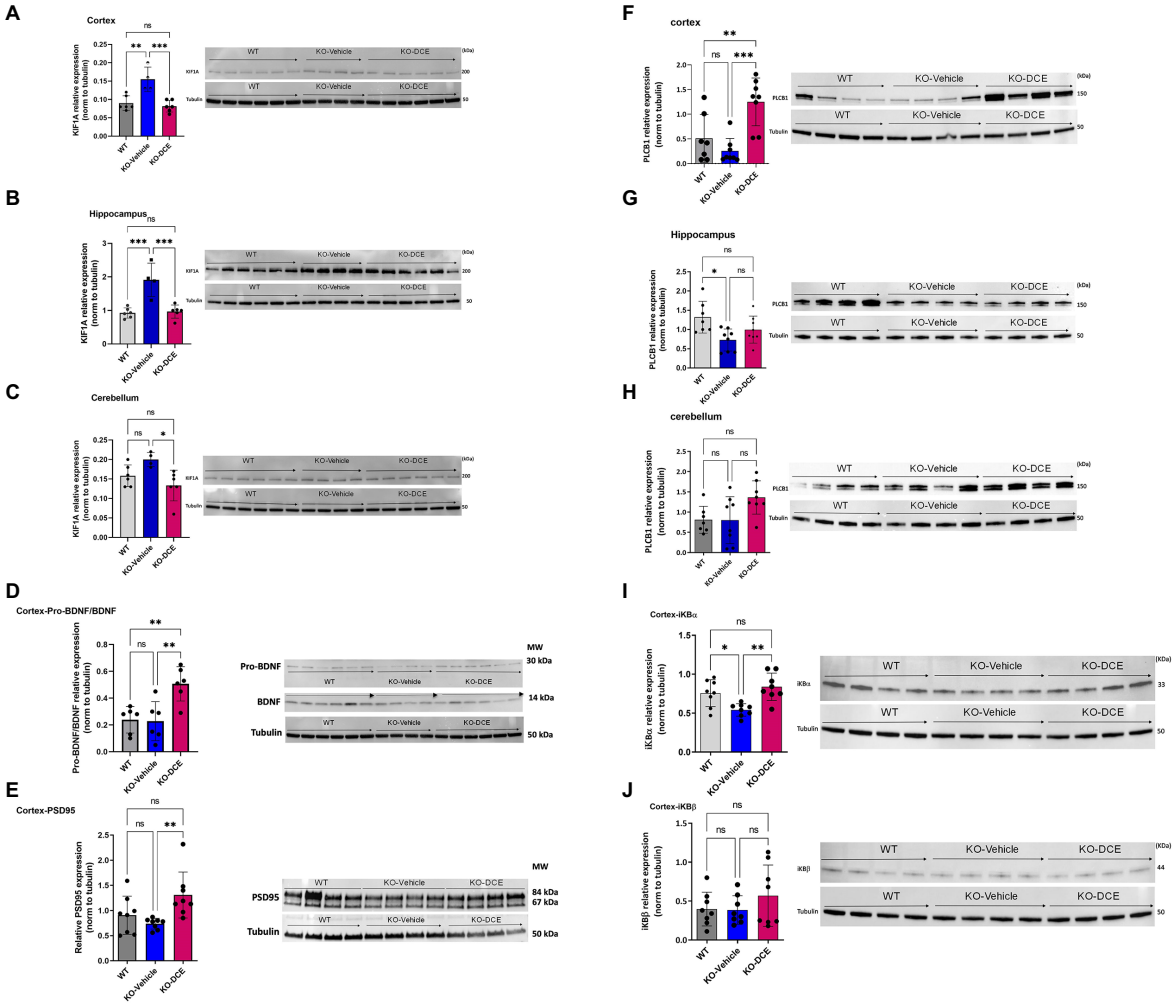
KIF1A and PLCB1 interplay is associated with DCE treatment efficacy in CrT Ko mice. **(A–C)** Western blot results showing that the KIF1A level was significantly increased in the cortex **(A)**, hippocampus **(B)** and cerebellum **(C)** in vehicle-treated CrT KO mice compared to WT mice, while DCE treatment rescued this overabundance in the three brain regions in CrT KO mice. **(D, E)** Western blot results showing that the pro-BDNF/BDNF ratio **(D)** was significantly altered in DCE-treated CrT KO mice compared to that of both WT mice and vehicle-treated CrT KO mice. In addition, the PSD95 level **(E)** was significantly altered in the cortices of vehicle-treated CrT KO mice compared to that of DCE-treated CrT KO mice. **(F–H)**, Western blotting showed that PLCB1 protein (150 kDa) abundance was increased in the cortex **(F)**, hippocampus **(G)** and cerebellum **(H)** in CrT KO mice at 0 days after DCE treatment. **(I,J)** Western blot results for Ibα **(I)** and IκBβ **(J)** showing that DCE promotes IκBα transcription factor abundance but not IκBβ transcription factor abundance. The data are the mean ± s.e.m. (*n* = 8/group). Statistical analysis was performed by one-way ANOVA followed by Tukey’s post-hoc test. Tubulin was used as a loading control. *p ≤ 0.05; **p ≤ 0.001; ***p ≤ 0.0001; ns = not significant.

KIF1A participates in vesicular transport, and vesicles containing the neurotrophin BDNF have been found to be under the control of KIF1A (Kondo et al., 2012). BDNF is produced in the neocortex throughout brain development and accelerates the overall redistribution of cortical neurons (Iki et al., 2005). DCE-mediated rescue of KIF1A levels in the cortex (Figure 4A), hippocampus (Figure 4B), and cerebellum (Figure 4C) of CrT KO mice resulted in higher levels of pro-BDNF/BDNF (*p* = 0.0043; Figure 4D) a long-term potentiation (LTP) biomarkers, which are linked to cognitive function improvement. These results indicate that KIF1A is a potential key player in CTD pathogenesis.

In order to highlight the central role of KIF1A abundance across the different brain regions of CrT KO mice, proteins co-immunoprecipitated together with KIF1A were identified by MS. Shotgun proteomics revealed that KIF1A and PLCB1 could be co-immunoprecipitated with anti-KIF1A antibody from cortical and hippocampal extracts (Supplementary Table 6). In addition, PLCB1 abundance was correlated with the DI in the object recognition test (Supplementary Table 5a) and spontaneous alternation rate in the Y-maze test (Supplementary Table 5b) (hippocampus: *r*^2^ = 0.460, *p* = 0.012; cortex: *r*^2^ = 0.469, *p* = 0.01, respectively). Therefore, further downstream functional analysis focused on KIF1A and PLCB1 proteins.

Additional analysis confirmed the abundance of PLCB1 by Western blotting (Figures 4F–H). The results showed that PLCB1 level decreased in the cortex (Figure 4F) and hippocampus (Figure 4G) in vehicle-treated CrT KO mice, while DCE-treated CrT KO mice showed a significant increase in PLCB1 levels in the cortex (*p* = 0.0004) compared to the hippocampus and cerebellum (Figure 4H).

DCE-mediated upregulation of PLCB1 levels in the cortex might might involved the NF-κβ pathway *via* dysregulation of a PKCα inhibitor, IκBα, thereby altering the abundance of NF-κβ-inducible genes. Thus, IκBα abundance was evaluated to determine whether the DCE-induced increase in PLCB1 levels affects targets downstream of this pathway. Western blotting indicated that IκBα (Figure 4I) (*p* < 0.05) but not IκBβ (Figure 4J) was significantly downregulated in the cortex in vehicle-treated CrT KO mice, while DCE treatment rescued IκBα protein levels in CrT KO mice (*p* = 0.008), suggesting that PLCB1 is involved in NF-κβ regulation in these mice.

## Discussion

The present study focuses on the CTD pathogenesis and the underlying mechanisms of DCE drug efficacy in CrT KO mouse model engineered to mimic the clinical features of CTD patients. Shotgun proteomics analysis, integrative bioinformatics and statistical modeling identified 14 key proteins that are dysregulated in the cerebellum, cortex, hippocampus and brain stem of *Slc6a8^−/y^* mice compared to WT mice and modulated by DCE. Notably, 13 of these proteins are related to ID disorders in human, including autism spectrum disorders (MECP2, Hammer et al., 2002; Shibayama et al., 2004; Wen et al., 2017), KIF1A (Weller and Gartner, 2001; Esmaeeli Nieh et al., 2015; Lee et al., 2015; Ohba et al., 2015; Padiath, 2016; Dai et al., 2017; Tomaselli et al., 2017), PLCB1 (Rusciano et al., 2021), NCAM1 (Arai et al., 2004; Atz et al., 2007; Gray et al., 2010; Varea et al., 2012), ANXA5 (Esmaeeli Nieh et al., 2015; Lee et al., 2015; Tomaselli et al., 2017), bipolar disorder (FABP7, Killoy et al., 2020), NCAM1, PLCB1 (Lo Vasco et al., 2013; Yang et al., 2016), axonal neuropathy (DCLK1), ID (KIF1A, Ohba et al., 2015), L1CAM (Weller and Gartner, 2001), regulation of neurite outgrowth (IGSF8, Ray and Treloar, 2012), leukodystrophy (LMNB1), cerebellar ataxia (LMNB1, Padiath, 2016; Dai et al., 2017), abnormal behavior (PI4KA, Pagnamenta et al., 2015), epileptic encephalopathies (PLCB1, MYO5A), or neurodegenerative diseases (ANK1, De Jager et al., 2014). Our findings suggest a profound alteration of the molecular landscape of the brain area of CrT KO mice, and DCE treatment changed a subset of those proteins. The hippocampus is crucial for spatial memory formation, while the Y-maze deficits are more rooted in the prefrontal cortex or the neocortex at the anterior end of the brain-than the hippocampus. The prefrontal cortex is an interconnected set of neocortical areas that have pattern of connectivity with all sensory neocortical and motor systems and a wide range of subcortical structures (Barnes and Pandya, 1992). The cerebellum is strongly interconnected with the cerebral hemispheres in both feedforward (cerebral hemispheres to cerebellum) and feedback directions (Schmahmann and Pandya, 1997). Since functional imaging and neuronal activation studies have shown that the sub-brain regions such as cortex, the cerebellum and the hippocampus are involved in the storage and retrieval of spatial memory and in the formation of spatial memory (Maviel et al., 2004), we were interested by the identification of subset of proteins correlated with the cognitive trajectory of CrT KO mice. Correlation analysis was performed using a stepwise regression model between the 14 selected proteins affected both by the mutation and DCE treatment.

**Figure 5:**
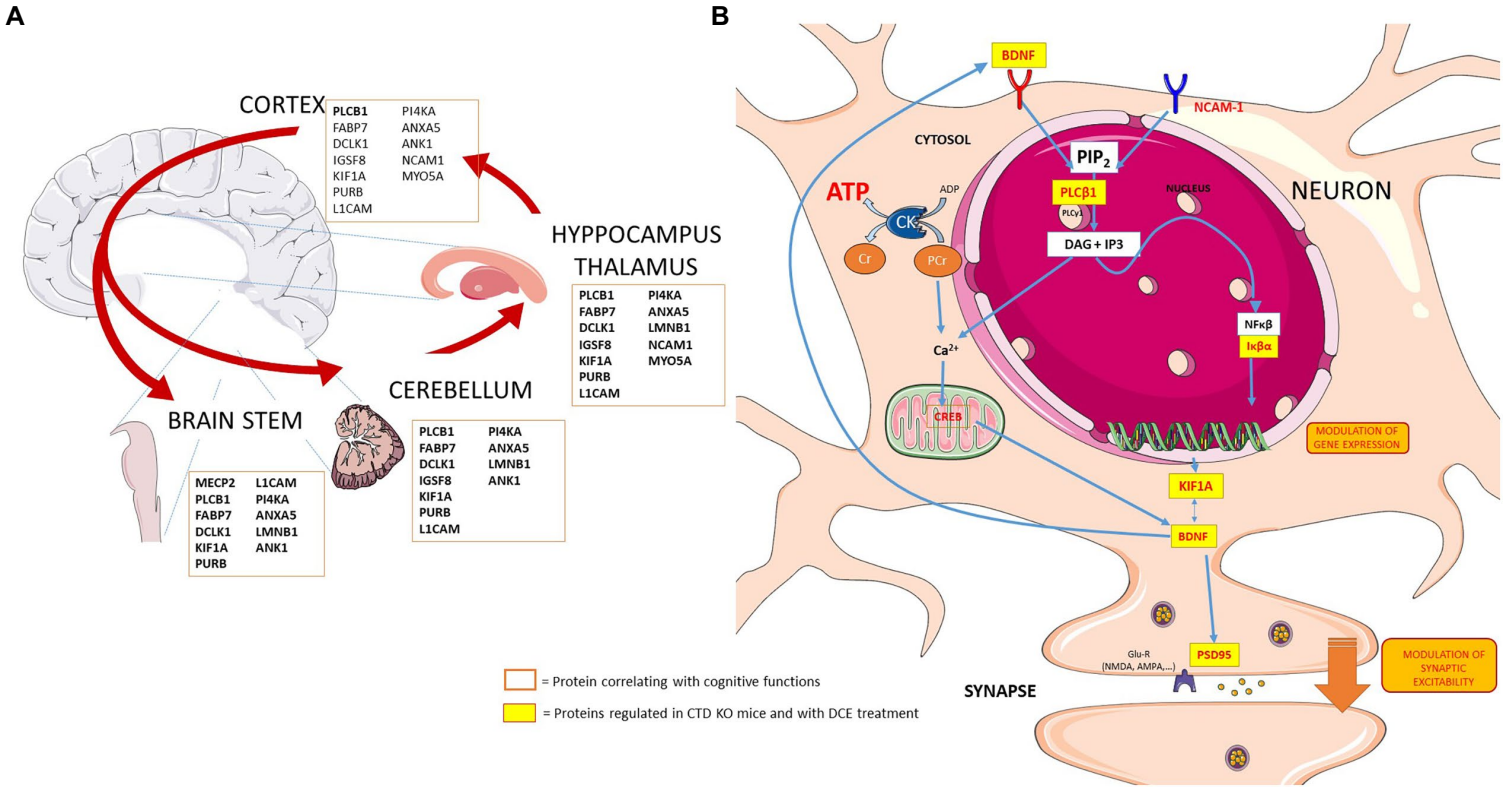
Schematic presentation showing the key players in the different brain regions and potential links between several proteins that are regulated in neurons in the context of CTD and by DCE treatment. **(A)** Proteins regulated by DCE in CrT KO mice. **(B)** DCE mediated the abundance of PLCB1. DCE-mediated upregulation of PLCB1 levels in the brain might lead to the production of inositol-1,4,5-triphosphate (IP3), and IP3 modulates the NF-κβ pathway *via* dysregulation of a PKCα inhibitor, IκBα, thereby altering the expression of NF-κβ-inducible genes which regulates the NF-κβ pathway by rescuing IκBα protein levels in CrT KO mice. NF-κβ is bound by IκBα and then translocates to the nucleus to activate target genes including KIF1A and BDNF.

Out of these 14 proteins, KIF1A abundance in the four brain regions (cortex, hippocampus, cerebellum and brain stem) was significantly correlated with DI in the object recognition test, while the abundance of this protein in the hippocampus, cortex and cerebellum correlated with the spontaneous alternation the Y-maze tests. Co-immunoprecipitation analysis using Western blotting confirmed that KIF1A interacts with PLCB1 in the various brain regions, suggesting a key role of KIF1A in CTD. The main observations of this study are summarized in Figure 5. These results confirm what has been reported in the literature regarding the association of KIF1A and PLCB1 to cognitive function in different brain disorders (Manning et al., 2012; Lee et al., 2015).

Novel object recognition test is a valid cognitive task focusing on mostly hippocampal function. CTD increased the abundance of KIF1A. These changes suggest that anteretrograde axonal transport may be impaired in hippocampal neurons which may lead to changes in synaptic proteins, thus contributing to changes in hippocampal neurotransmission and to cognitive and memory impairments. In a rat model of α-synucleinopathy, elevated levels of KIF1A were observed in substantia nigra (Chung et al., 2009), suggesting the possibilily of the imbalance in protein degradation and synthesis and/or axonal transport deficit. KIF1A mutations have been found in patients with a severe neurodevelopmental disorder with Rett syndrome patients (Wang et al., 2019). KIF1A has been found to participate in vesicular transport. Vesicles containing the neurotrophin BDNF have been found to be under the control of KIF1A (Kondo et al., 2012). BDNF is produced in the neocortex throughout brain development and accelerates the overall redistribution of cortical neurons. DCE-mediated rescue of KIF1A levels in CrT KO mice resulted in higher levels of pro-BDNF/BDNF, which are linked to cognitive function improvement, suggesting that normal expression of KIF1A but not overexpression might be indispensable for BDNF-mediated cognitive function. The direct effect of DCE on the regulation of pro-BDNF/BDNF expression could not be excluded. KIF1A overabundance in the developing brains of CrT KO mice likely leads to synaptic dysfunction, thus contributing to cognitive and memory impairments (Iki et al., 2005; Lu et al., 2014). This is in agreement with previous data showing the reduced cortical spine density and reductions in protein levels of several synaptic markers in the brains of Slc6a8−/y mice (Chen et al., 2021). In addition, we found that in the cortex, the abundances of the presynaptic protein IgSF8 which has been reported to be a critical regulator of brain microcircuits and neuronal function (Apostolo et al., 2020), was altered in vehicle-treated CrT KO mice compared with the abundances in WT mice. Y-maze exploits the inherent motivation of an organism to explore an unknown environment. This assay is very sensitive for assessing working memory and cognitive flexibility in mice providing a simple and widely applicable behavioural assay with exceptional translational relevance. Many parts of the brain--including the hippocampus and prefrontal cortex--are involved in this task (Maviel et al., 2004). We noticed the abundance of KIF1A across the hippocampus and cortex in CrT KO mice correlating with the cognitive performance in Y-maze test. These findings indicate that KIF1A is one of a potential key player in CTD pathogenesis.

Data from co-ip experiments indicated in Supplementary File S11 suggest that it is likely a transient complex has been formed between KIF1A and PLCB1. PLCB1 plays a major role in vesicular trafficking within the cell, thus playing a direct role in axonal transport of neurotransmitters (Hannan et al., 2001). PLCB1 dysregulated signaling is linked to several brain disorders, including epilepsy, schizophrenia, bipolar disorder, Huntington’s disease, depression and Alzheimer’s disease (Alberini, 2009; Cholewa-Waclaw et al., 2016). We demonstrated that DCE modulates the relative abundance of PLCB1 in different brain regions. Our data showed that PLCB1 regulates the NF-κβ pathway by rescuing IκBα protein levels in CrT KO mice. Of note, NF-κβ signaling in the brain has been implicated in regulating neuronal survival and function (Meffert et al., 2003; Kaltschmidt et al., 2006). NF-κβ is bound by IκBα and then translocates to the nucleus to activate target genes. IκBα-deficient mice display deregulated and sustained NF-κβ activation (Lian et al., 2012), indicating a critical role for IκBα in NF-κβ regulation. CrT KO mice showed a decrease in IκBα abundance in the cortex and hippocampus, which was rescued by DCE treatment. It seems likely, DCE-mediated upregulation of PLCB1 levels in the cortex might lead to the production of inositol-1,4,5-triphosphate (IP3) (Rusciano et al., 2021), and IP3 modulates the NF-κβ pathway *via* dysregulation of a PKCα inhibitor, IκBα, thereby altering the abundance of NF-κβ-inducible genes. The decrease in IκBα abundance might regulates neuroinflammation as well as spatial memory formation and synaptic plasticity, probably through BDNF signaling. Our findings are in agreement with previous observations showing that stimulation of the neuroinflammatory response through NF-κβ activation may be therapeutically beneficial (Lian et al., 2012). The regulation of neuroinflammation is also supported by the identification of NCAM1 in CrT KO mice whose abundance was altered and restored by DCE treatment; NCAM1 could participates in the structural deficits in CrT Ko mice including changes in neuronal migration and synaptogenesis.

In conclusion, the results of the study indicated that the crosstalk between KIF1A and PLCB1 mediates cognitive function in the CTD. In addition, the results identified a panel of additional 12 proteins which suggests that Slc6a8−/y mice could have structural deficits including changes in neuronal migration or synaptogenesis. To our knowledge, this is the first study that describes some of the molecular mechanisms of CTD-related cognitive dysfunction and the therapeutic effect of DCE. Furthermore, the results of the study provide further evidence regarding the efficacy of DCE in treating the cognitive symptoms of CTD and restoring the abundance of key molecular players to normal levels in several brain regions. Little is known about the underlying mechanisms of the Cr-mediated behavioral deficits. Correlating proteomic changes to behavioral deficits provide mechanistic insights into Cr-mediated changes. Future studies can be designed to investigate this relationship. This study provides a shift in research paradigms and an advancement in intervention for CTD. While CTD carrier females are reported to have a milder phenotype, it will be of interest in the future to assess the potential alteration of the molecular landscape of the brain area of for females that have a milder phenotype.

## Materials and methods

### Ethical considerations

All *in vivo* experiments were conducted in compliance with the European Communities Council Directive of 22 September 2010 and were approved by the Italian Ministry of Health (authorization number 259/2016-PR).

### Generation of CrT KO mice

Male CrT^−/y^ and CrT^+/y^ mice were generated on the C57BL/6 J background as previously described (Baroncelli et al., 2014). The mice were housed at 22°C on a 12–12 h light–dark cycle and provided food and water *ad libitum*.

The presence of the *Slc6a8* mutation was confirmed by PCR as previously described (Baroncelli et al., 2016). Briefly, genomic DNA was isolated from tail tissue collected from P25 mice using the DNeasy^®^ Blood & Tissue kit from Qiagen according to the manufacturer’s protocol. The following primers were used for PCR amplification: F: AGGTTTCCTCAGGTTATAGAGA; R: CCCTAGGT GTATCTAACATCT; R1: TCGTGGTATCGTTATGCGCC. The amplicon sizes were as follows: CrT^+/y^ allele = 462 bp; mutant allele = 371 bp.

### DCE treatment

DCE was prepared as previously described (Trotier-Faurion et al., 2013). Ten milligrams of DCE was added to 0.375 g of Maisine®CC (Gattefossé) at room temperature, and then 125 mg of DHA (Sigma–Aldrich) was added. The mixture was vortexed for 5 min and shaken at 1,000 × g in a thermomixer at 30°C for 48 h. Then, the sample was centrifuged at 20,000 × g for 10 min at room temperature, and the resulting supernatant was filtered through a 0.22 μm filter, placed in another tube and stored at +4°C prior to use.

DCE was intranasally administered to CrT KO mice for 30 days as previously reported (Ullio-Gamboa et al., 2019), while wild-type (WT) and vehicle-treated mice were used as controls (*N* = 8/group). 6 μL of DCE or vehicle (Maisine®CC with DHA) was placed in the nostril. The DCE (4 mg/g) or vehicle was given twice bilaterally (12 μL total volume).

### Behavioral testing

Behavioral testing started 14 days after the start of the treatment as was done previously (Baroncelli et al., 2016). Treatment continued during behavioral testing, which lasted 2 weeks, for a total of 30 days of treatment. Each mouse was subjected to all of the behavioral assessments in following order: the 24-h ORT (3 days), Y-maze test (1 day), and hidden platform MWM test (7 days).

#### ORT

The ORT apparatus consisted of a square arena (60 × 60 × 30 cm) made of polyvinyl chloride with black walls and a white floor as previously described (Baroncelli et al., 2016). The day before testing, the mice were individually habituated to the empty arena for 10 min. The ORT, which is based on the tendency of rodents to spend more time exploring a novel object than a familiar object, was used to measure short-and long-term memory and consisted of the sample phase and testing phase. During the sample phase, two identical objects were placed in diagonally opposite corners of the arena (approximately 6 cm from the walls), and the mice were allowed to explore the arena for 10 min. The testing phase was performed 24 h after the sample phase. An identical copy of one of the objects from the sample phase and a novel object were placed in the same locations, and the mice were returned to the arena and allowed to explore the objects for 5 min. The DI was calculated as follows: DI = (T new − T old)/(T new + T old), where T new is the time spent exploring the novel object and T old is the time spent exploring the familiar object (Baroncelli et al., 2016).

#### Y-maze spontaneous alternation test

The spontaneous alternation rate was measured using a Y-shaped maze with three symmetrical gray solid plastic arms at a 120-degree angle (26 cm long, 10 cm wide, and 15 cm high) as previously described (Begenisic et al., 2014; Baroncelli et al., 2016). The mice were placed in the center of the maze one at a time, and their movements were recorded for 8 min. The number of arm entries (all four limbs within an arm) and the number of triads (successive entries into all three arms) were recorded to calculate the spontaneous alternation percentage (defined as the number of triads divided by the number of possible alternations (total arm entries minus 2) multiplied by 100).

#### MWM test

The mice were subjected to 4 training trials per day for a total of 7 days. The apparatus consisted of a circular water tank (diameter, 120 cm; height, 40 cm) filled with water (23°C) to a depth of 25 cm. The water was made opaque by the addition of nontoxic white paint. Four starting positions arbitrarily designated the north (N), south (S), east (E), and west (W) positions were selected, thus dividing the tank into 4 quadrants. A square escape platform (11 × 11 cm) was submerged 0.5 cm below the water surface in the middle of one of the 4 quadrants. The mice were allowed to search for the escape platform for up to 60 s, and their swimming paths were automatically recorded by the Noldus Ethovision system. The last trial on the last training day was a probe trial, during which the escape platform was removed from the tank and the swimming paths of the mice were recorded for 60 s while they searched for the missing platform.

### Proteolysis and MS

Before MS analysis, total protein (15 μg) was extracted from tissues from the five tissues, i.e., muscle, cortical, cerebellar, hippocampal and brainstem tissues, then mixed with lithium dodecyl sulfate lysis buffer (Invitrogen), and incubated at 99°C for 5 min. Samples were then separated by electrophoresis for a short amount of time (5 min) at 200 V on a NuPAGE 4–12% Bis-Tris gel in 1X MES/SDS (Invitrogen) running buffer. The gels were stained with SimplyBlue SafeStain (Thermo) for 5 min followed by an overnight wash in water with gentle agitation. The band containing the whole proteome from each sample was excised from the polyacrylamide gel and treated as previously described (Hartmann and Armengaud, 2014). The proteins were in-gel proteolyzed with trypsin gold (Promega) in the presence of 0.01% Protease Max surfactant (Promega) at 50°C for 60 min. A total of 1 μL of the resulting peptide fraction (50 μL), corresponding to approximately 300 ng of peptide, was analyzed by liquid chromatography–tandem mass spectrometry (LC–MS/MS) using an Ultimate 3,000 nano-LC system coupled to a Q-Exactive HF mass spectrometer (Thermo Scientific) as described previously (Hartmann and Armengaud, 2014). The peptides were loaded on a reverse-phase PepMap 100 C18 μ-precolumn (5 μm, 100 Å, 300 μm i.d. × 5 mm, Thermo Fisher) and then resolved on a nanoscale PepMap 100 C18 nanoLC column (3 μm, 100 Å, 75 μm i.d. × 50 cm, Thermo Fisher) at a flow rate of 0.2 μL.min^−1^ using a 90-min gradient (4% B from 0 to 3 min, 4–25% B from 3 to 78 min and 25–40% B from 78 to 93 min), with 0.1% HCOOH/100% H_2_O as mobile phase A and 0.1% HCOOH/80% CH3CN/20% H_2_O as mobile phase B. The mass spectrometer was operated in Top20 mode, with a scan range of 350 to 1800 *m/z*, and selection and fragmentation were performed using a 10 s dynamic exclusion time for the 20 most abundant precursor ions. Only ion precursors with a 2+ or 3+ charge were selected for HCD fragmentation, which was performed at a normalized collision energy of 27 eV.

### MS/MS spectra interpretation and differential proteomics

MS/MS spectra were assigned using Mascot Daemon software version 2.6.1 (Matrix Science) and the *Mus musculus* SwissProt database comprising 17,096 protein sequences. Peptide tolerance, MS/MS fragment tolerance, and the maximum number of missed cleavages were set to 5 ppm, 0.02 Da and 2, respectively. Carbamidomethylation of cysteine was considered a fixed modification, and oxidation of methionine was considered a variable modification. Peptides with *p* value ≤0.05 for homology threshold mode and proteins with at least two distinct peptides were selected (false discovery rate < 1%).

### Bioinformatics analysis of proteomics data

The general workflow of the bioinformatics analysis is shown in Supplementary Figure 1. An in-house script was written using the R programming language to identify differentially expressed proteins between the three groups (WT, vehicle-treated CrT KO mice and DCE-treated CrT KO mice) in each of the five tissues, i.e., muscle, cortical, cerebellar, hippocampal and brainstem tissues. The following comparisons were analyzed: vehicle-treated CrT KO mice vs. WT mice; DCE-treated CrT KO mice vs. vehicle-treated CrT KO mice; and DCE-treated CrT KO mice vs. WT. Initially, the proteomics data were normalized using variance stabilizing normalization (VSN, Motakis et al., 2006). Proteins with at least 10 assigned MS/MS spectra across all samples were retained. An unsupervised variation filter was then applied to the proteomics data (Hamoudi et al., 2010), and samples of 8 proteins with MS/MS spectra were included. Differential abundance analysis of proteins among the different regions was carried out using a modified R package for reproducibility-optimized statistical testing (ROTS, Suomi et al., 2017). The data were sorted according to the adjusted p value based on a false discovery rate < 0.05. Reproducibility plots and principal component analysis (PCA) were used to assess the quality of the separation of the data between the various groups that were being compared. The identified differentially expressed proteins were visualized using volcano plots and heatmaps. The heatmaps were generated using unsupervised hierarchical clustering carried out on the basis of Ward linkage and Euclidean distance to assess the degree of proteomic profile separation across the four brain regions among the three groups.

### Pathway analysis

Pathway analysis was performed to narrow down the differentially abundant proteins and identify their potential functions. To achieve this, the proteins whose abundances were significantly altered in CrT KO vehicle-treated mice compared with WT mice and in DCE-treated mice compared with vehicle-treated mice were selected for subsequent pathway analysis. The proteins whose abundances were altered in vehicle-treated mice and DCE-treated mice compared with WT mice were also selected. Pathway analysis using gene set enrichment was carried out using Enrichr (Chen et al., 2013; Kuleshov et al., 2016) focusing on the following sets: BioCarta_2016, Elsevier_Pathway_Collection, GO_Biological_Process_2018, GO_Molecular_Function_2018, KEGG_2019_Human, KEGG_2019_Mouse, MSigDB_Hallmark_2020, WikiPathways_2019_Mouse, WikiPathways_2019_Human, ClinVar_2019, DisGeNET, Jensen_DISEASES, and OMIM_Disease. Relevant pathways were selected based on a cutoff of *p* < 0.05. The set of proteins found to be significantly involved in the different pathways and diseases were selected for further analysis.

### Statistical analysis of the differentially expressed proteomics data

In order to identify the patterns of differentially expressed proteins across the different regions, the data identified by ENRICHR analysis was used to construct a multivariate statistical model using one-way ANOVA followed by Bonferroni’s *post hoc* test for comparisonsacross the cortex, cerebellum, brainstem, hippocampus and muscle. The muscle was used as control for tissue other than the brain.

The stepwise regression statistical modelling was used to further reduce the marker set and identify the proteins whose abundances were significantly altered by the mutation and restored by the DCE treatment. To identify the proteins involved in cognition, a stepwise regression model was constructed to assess the correlation between the levels of the differentially expressed proteins and performance in the ORT (DI) and the Y-maze test. The results were further validated using Pearson correlation analysis of the differentially abundant proteins among the different groups.

### Western blotting

Since PLCB1 and KIF1A abundance as well as their partners are associated to several brain disorders, Western blotting was used to determine their abundance as well as two inhibitors of NF-κB, IκBα and IκBβ, in dissected brain tissues. Briefly, brain tissues were homogenized in freshly prepared lysis buffer containing 20 mM Trizma-Base, 150 mM NaCl (pH 7.4) (Sigma–Aldrich, Saint-Quentin Fallavier, France), 1% Triton X-100, 4% complete protease inhibitor cocktail and 20% mix of anti-phosphatase inhibitors using a Precellys Evolution tissue homogenizer. The samples were then centrifuged at 2500 × g for 15 min followed by 10,000 x g for 20 min to obtain lysates for electrophoresis. The proteins (10 to 20 μg) and protein standards were mixed with Laemmli buffer and loaded on 4–15% Criterion TGX Stain-Free protein gels in 1 × TGS running buffer (all from Bio-Rad, Marnes-la-Coquette, France) and transferred to a 0.2 μm PVDF membrane with the Trans-Blot Turbo RTA Midi Transfer Kit (Bio-Rad, Marnes-la-Coquette, France). The membranes were blocked for 30 min in 5% low-fat milk in TBS-0.1% Tween 20 at room temperature. The blots were probed with specific primary antibodies overnight at 4°C followed by horseradish peroxidase (HRP) secondary antibodies diluted 1:5000 or 1:50000 in 5% low-fat milk in TBS-0.1% Tween 20 at room temperature. For protein detection, the membranes were treated with ECL Prime Western Blotting reagent (Amersham, UK) or Clarity Western ECL Substrate and exposed with a ChemiDoc Touch Imaging System (Bio-Rad, Marnes-la-Coquette, France). The band density was quantified with Image Lab software (Bio-Rad, Marnes-la-Coquette, France). The following antibodies were used at the indicated dilutions: anti-PLCB1 (1:1000, Abcam, ab182359), anti-IκBα (1:500, Cell Signaling Technology, 4812S), anti-IκBβ (1:500, Cell Signaling Technology, 15519S), anti-PSD95 (1:2000, Merck, MABN68), anti-tubulin (1:2000, Sigma–Aldrich, T6199), and anti-KIF1A (1:1000, Abcam, ab180153).

### Co-IP

Protein extract samples (200 μg) from cortex and hippocampus were adjusted to a final volume of 600 μL with binding buffer (20 mM Tris–HCl (pH 7.5), 150 mM NaCl, 10% glycerol, 1 mM EDTA, 0.1% BSA, and 1X protease inhibitor) before the addition of 17.6 μL anti-KIF1A antibody and 100 U of Benzonase nuclease (Novagen 70,746–3). The mixture was incubated overnight at 4°C on a rotating wheel. Forty-three microliters of Dynabeads protein G (Invitrogen, 10003D) were washed 3 times with PBS + 0.05% Tween and once with binding buffer. The beads were then added to the immunoprecipitate and incubated for 1 h at room temperature with rotation. After incubation, the immunoprecipitate was washed twice with Benzonase buffer (20 mM Tris–HCl (pH 8.0), 20 mM NaCl, 10% glycerol, 2 mM MgCl2, 0.1% BSA, and 1X protease inhibitor (Roche)) and incubated in Benzonase buffer supplemented with 100 U of Benzonase nuclease for 30 min at 37°C before being washed three times with washing buffer (20 mM Tris–HCl (pH 7.5), 150 mM NaCl, 10% glycerol, 1 mM EDTA, 0.05% Tween, and 1X protease inhibitor). The immunoprecipitated proteins were eluted directly in 25 μL 1.5× Laemmli buffer supplemented with 200 mM DTT and 1 mM beta-mercaptoethanol at 95°C for 10 min before magnetic separation of the beads and MS. MS was carried out under similar conditions as those for the brain protein extracts except that the nano-UPLC gradient was reduced to 60 min.

## Data availability statement

The MS and proteomics dataset is available through the ProteomeXchange Consortium via the PRIDE partner repository (https://www.ebi.ac.uk/pride/) under dataset identifiers PXD024968 and 10.6019/PXD024968.

## Ethics statement

The animal study was reviewed and approved by All *in vivo* experiments were conducted in compliance with the European Communities Council Directive of 22 September 2010 and were approved by the Italian Ministry of Health (authorization number 259/2016-PR).

## Author contributions

AM was responsible for project administration, conceptualization, funding acquisition and writing of the manuscript. FC and LB administered drugs to the mice, conducted behavioral studies, and collected brain samples. EP administered drugs to the mice and performed genotyping and the behavioral studies. AC-G, LBB, and NC conducted biochemistry experiments. EM and CS conducted the Co-IP experiments. J-CG and JA designed and conducted the proteomic experiments. RaH and RiH conducted mathematical and statistical modeling of the proteomics data as well as bioinformatics analysis. MS, RaH, LB, JA, RiH, HB, and TJ participated in the writing and review of the manuscript. All authors contributed to the article and approved the submitted version.

## Funding

This work was supported by Jerome Fondation Lejeune grant and by X-traordinaire, which is a patient group dedicated to rare intellectual disabilities. AM is funded by Foundation Lejeune grant; RiH is funded by the University of Sharjah - Skotech collaborative Artificial Intelligence for Life (AIfoL) award (grant no: AIfoL-2201).

## Conflict of interest

The authors declare that the research was conducted in the absence of any commercial or financial relationships that could be construed as a potential conflict of interest.

## Publisher’s note

All claims expressed in this article are solely those of the authors and do not necessarily represent those of their affiliated organizations, or those of the publisher, the editors and the reviewers. Any product that may be evaluated in this article, or claim that may be made by its manufacturer, is not guaranteed or endorsed by the publisher.
