# Supplementary material for: Dodecyl creatine ester improves cognitive function and identifies key protein drivers including KIF1A and PLCB1 in a mouse model of creatine transporter deficiency
Source: Front Mol Neurosci. 2023 Mar 24;16:1118707. doi: 10.3389/fnmol.2023.1118707 (PMC10103630; doi:10.3389/fnmol.2023.1118707)
Supplement: Supplementary file 1 [file Data_Sheet_1.zip › Supplementary Material data sheet 1/Legends of Supplementary Material data sheet 1.docx]

**Supplemental Fig. 1: Flowchart depicting the workflow used to identify the key proteins that are associated with cognitive functions and regulated by DCE treatment in CTD pathogenesis.**

**1.**The raw data of 4035 proteins was generated using Q-exactive HF mass spectrometer. **2.** The raw proteomics data were normalized using the variance stabilizing normalization (VSN) function (Supplemental Table 1), and an unsupervised filter was applied. **3a.** The differentially abundant proteins between the three groups (WT, vehicle-treated CrT KO mice and DCE-treated CrT KO mice) in each brain region were identified using reproducibility-optimized statistical testing (ROTS) and sorting according to the adjusted p-value based on False Discovery Rate. **3b.** The quality of the separation of the data between the various groups being compared was assessed using reproducibility plots and PCA. The differentially expressed proteins were visualized using volcano plots (Supplemental Fig. 2a-d). The degree of separation between the groups was assessed using unsupervised hierarchical clustering (Supplemental Fig. 3a-h). **4.** Venn diagram of the differentially expressed proteins was generated, and the overlapping proteins that showed a significant change in abundance in CrT KO mice compared with WT mice and in DCE-treated mice compared with vehicle-treated mice were selected for subsequent pathway analysis (Fig. 2a-d & Supplemental Table 2). Pathway analysis of the overlapping proteins was performed using Enrichr with a cutoff value of p< 0.05, and the proteins found to be involved in the different pathways and diseases were selected for further analysis. **5.** To identify the patterns of differentially expressed proteins across the different regions, the data of the proteins selected from the pathway analysis were used to construct a multivariate statistical model using one-way ANOVA followed by Bonferroni’s post hoc test for comparisons among the cortex, cerebellum, brainstem, hippocampus and muscle (Fig. 2e-f). **6.** The proteins whose abundances were most markedly altered by the mutation and DCE treatment were selected. Those most abundant proteins were significantly altered by the mutation compared to WT (vehicle-treated vs WT) and by the treatment compared to vehicle (DCE-treated vs. vehicle treated) and their abundance after treatment was restored to levels comparable to those in WT mice (Fig. 3a; Supplemental Tables 3 & 4). **7.** To identify the proteins involved in cognition, a stepwise regression model was constructed to assess the correlation between the levels of the differentially expressed proteins and performance in the ORT (DI) and the Y-maze test (Fig. 3b-c and Supplemental Table 5).

**Supplemental Fig. 2. Reproducibility plots, PCA and volcano plots for the four brain regions evaluated.**

**a-d**, The differentially expressed proteins in the four brain regions (cortex (**a**), cerebellum (**b**), brainstem (**c**), and hippocampus (**d**)) were visualized using volcano plots, and the quality of the separation of the data between the various groups being compared (vehicle-treated CrT KO mice vs. WT mice) and (DCE-treated vs. vehicle-treated CrT KO mice) was assessed using reproducibility plots and PCA.

**Supplemental Fig. 3. Unsupervised hierarchical clustering in the four brain regions.**

The degree of separation among the groups (vehicle-treated CrT KO mice, DCE-treated CrT KO mice, and WT mice) in the four brain regions (cortex (**a, b**), cerebellum (**c, d**), brainstem (**e, f**), and hippocampus (**g, h**)) was assessed using unsupervised hierarchical clustering. The proteins shown on the clusters are listed in order in the table associated.

**Supplemental Table 6: Results of shotgun analysis of anti-KIF1A immunoprecipitates**
