# Supplementary figures and images for: Dodecyl creatine ester improves cognitive function and identifies key protein drivers including KIF1A and PLCB1 in a mouse model of creatine transporter deficiency
Source: Front Mol Neurosci. 2023 Mar 24;16:1118707. doi: 10.3389/fnmol.2023.1118707 (PMC10103630; doi:10.3389/fnmol.2023.1118707)

**Cortex**

**Veh vs WT**


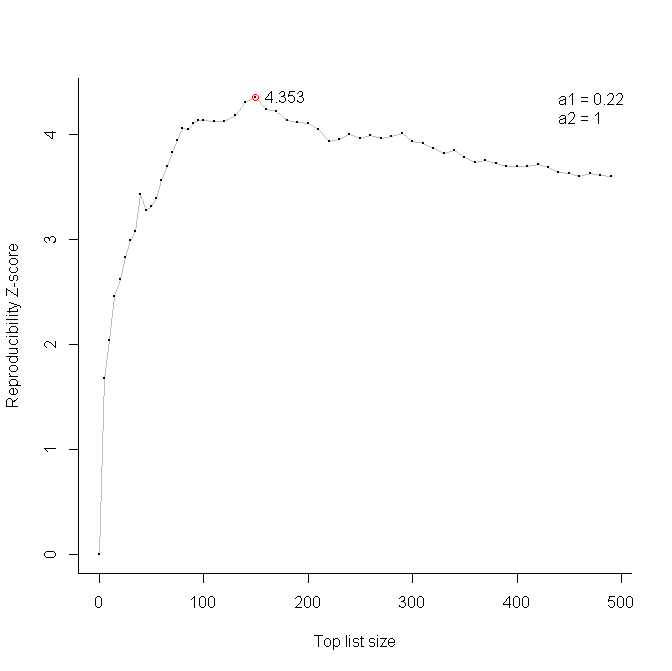


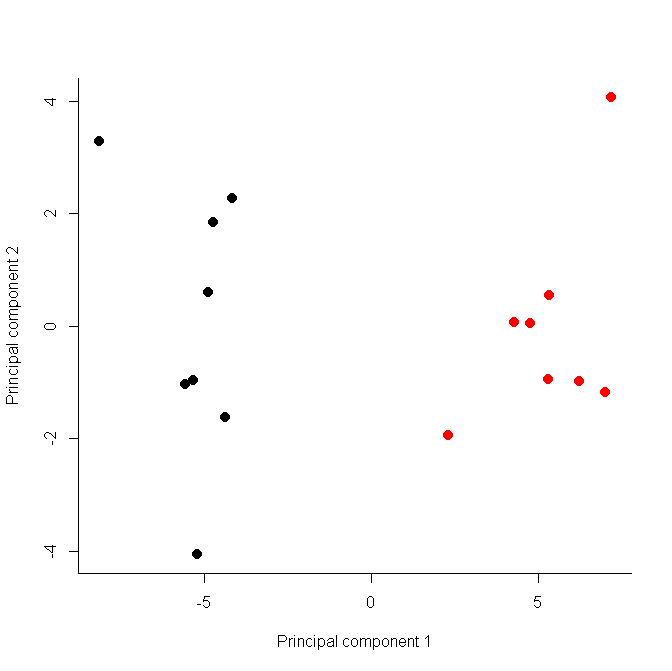


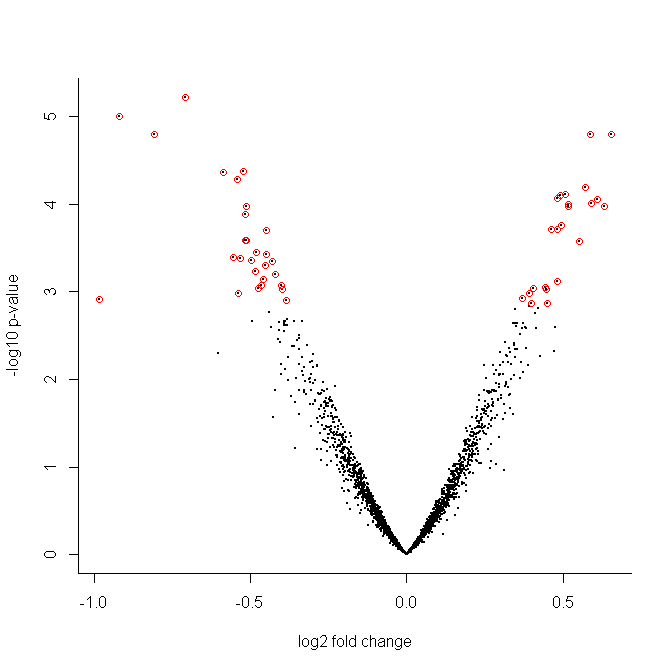


**Cortex**

**DCE vs Veh**


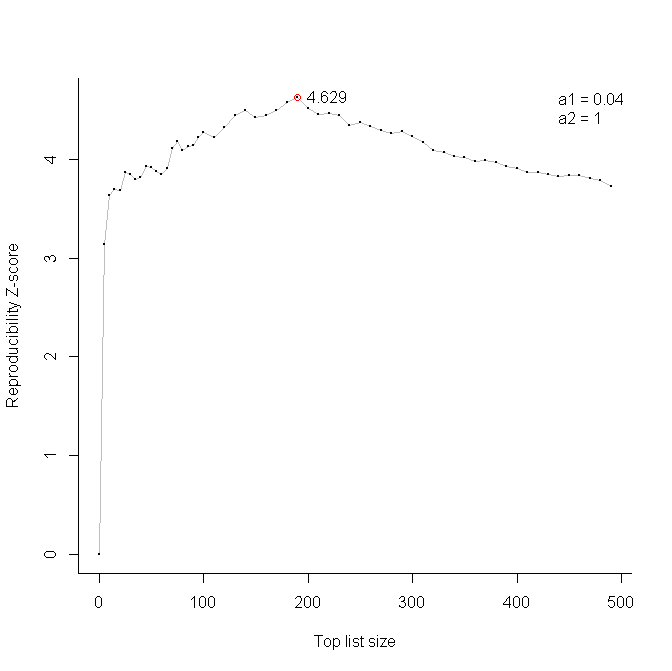


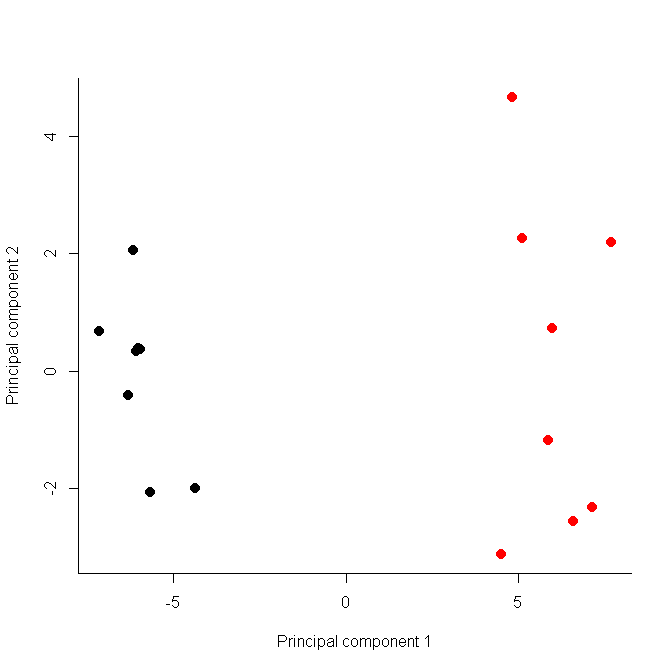


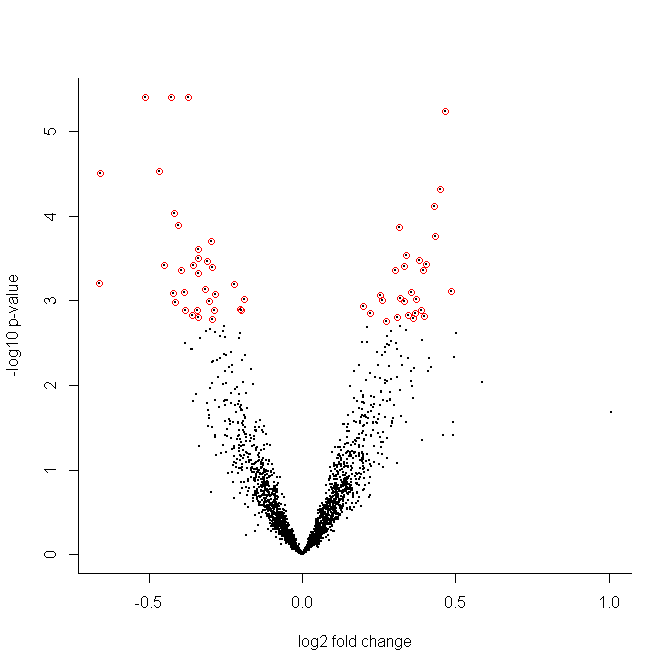

Supplement: Supplementary file 1 [file Data_Sheet_1.zip › Supplementary Material data sheet 1/Suppl fig 2a.docx]

**Cerebellum**

**Veh vs WT**
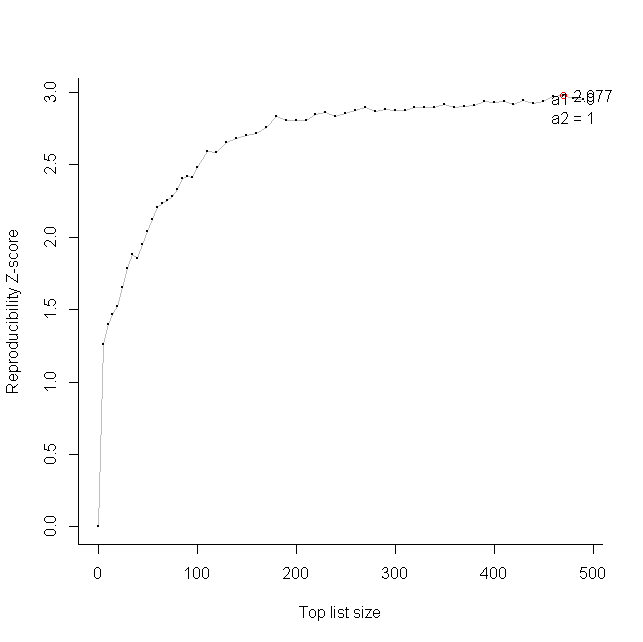

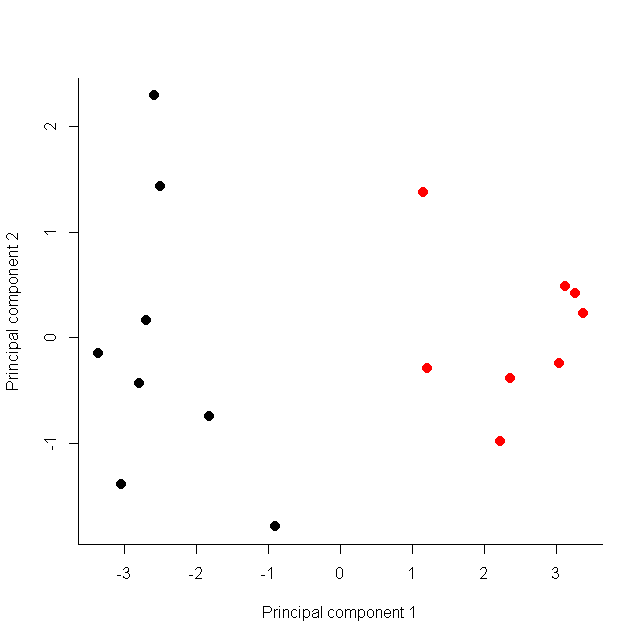

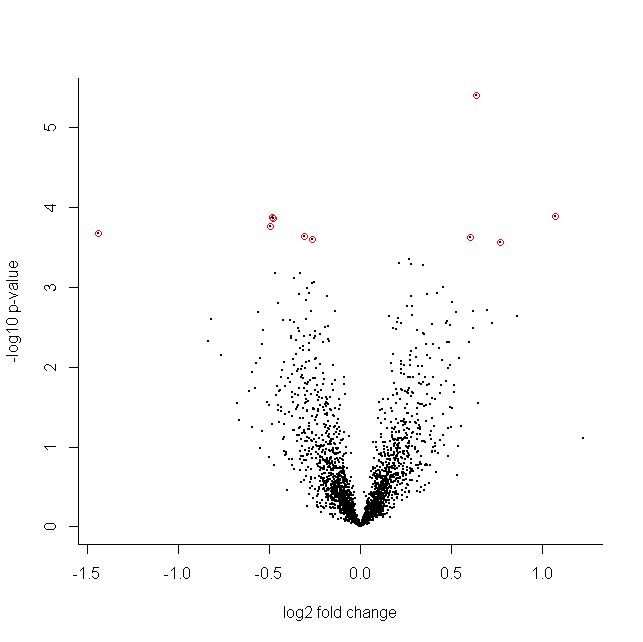


**Cerebellum**

**DCE vs Veh**
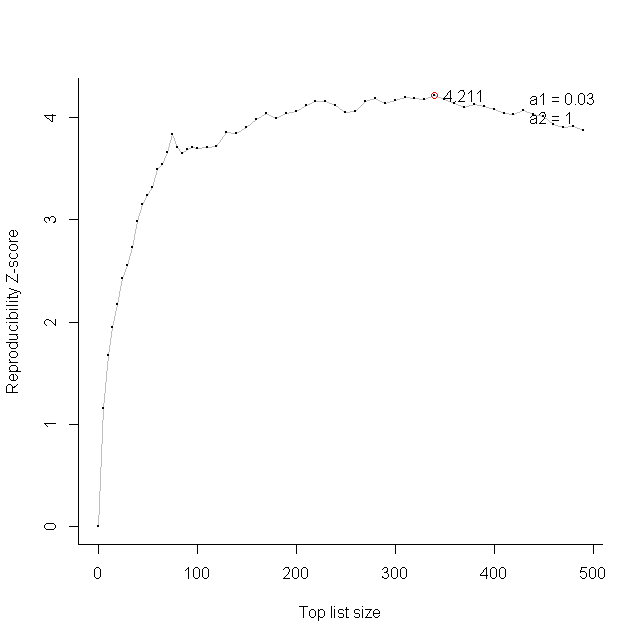

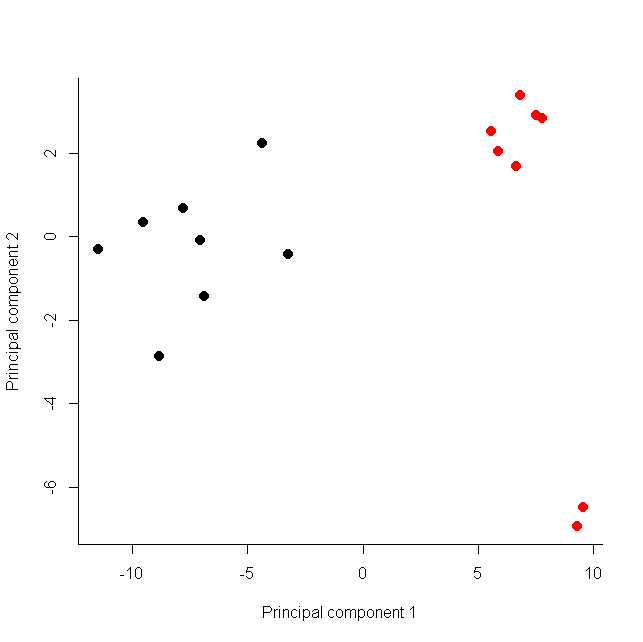

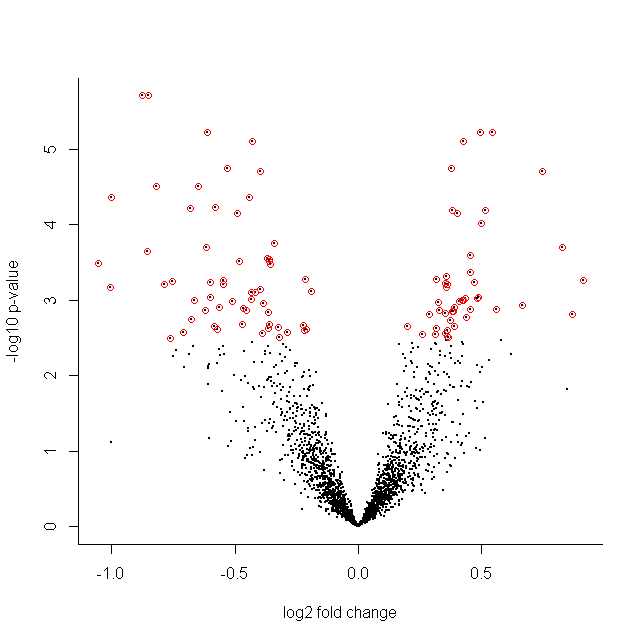

Supplement: Supplementary file 1 [file Data_Sheet_1.zip › Supplementary Material data sheet 1/Suppl fig 2b.docx]

**Brain Stem**

**Veh vs WT**


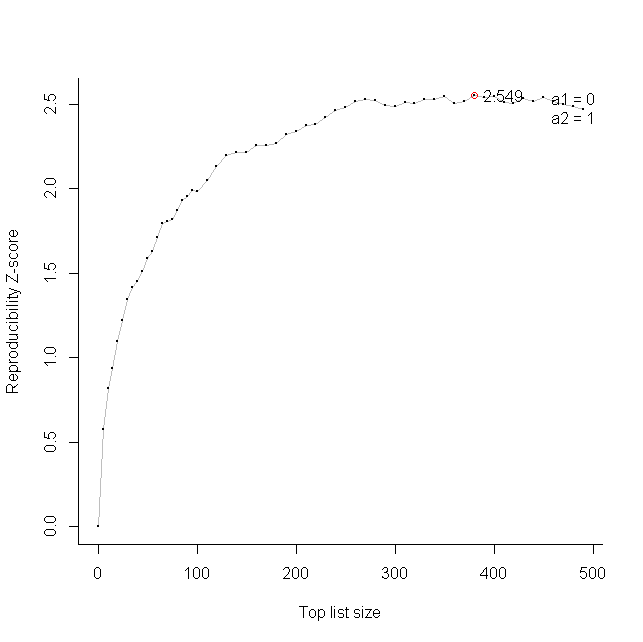


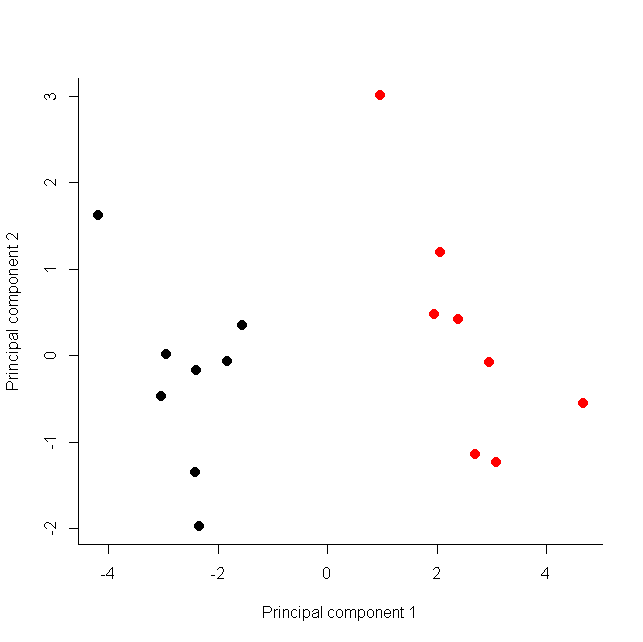

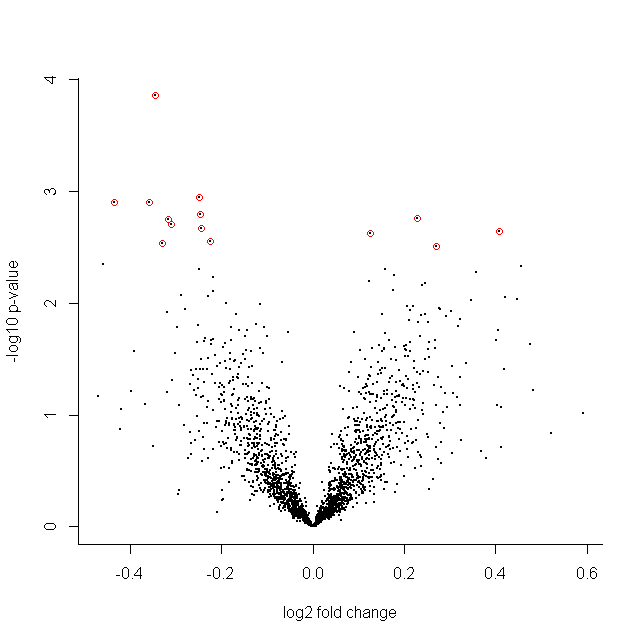


**Brain Stem**

**DCE vs Veh**


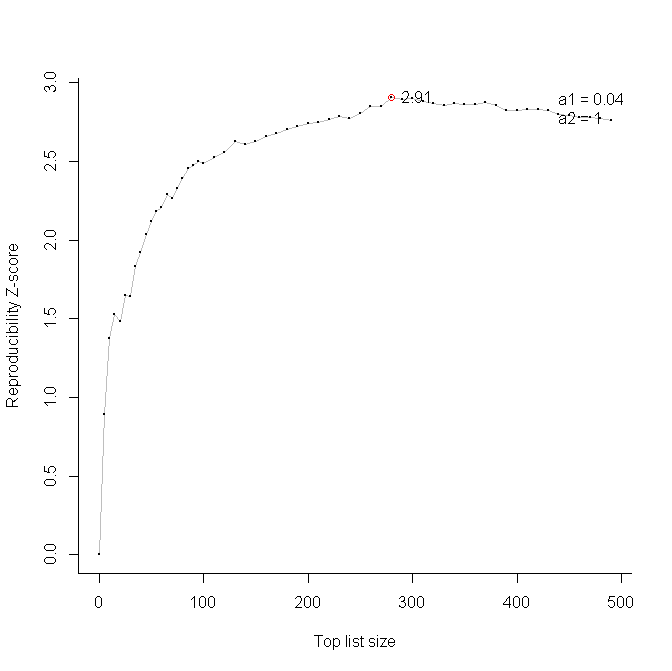


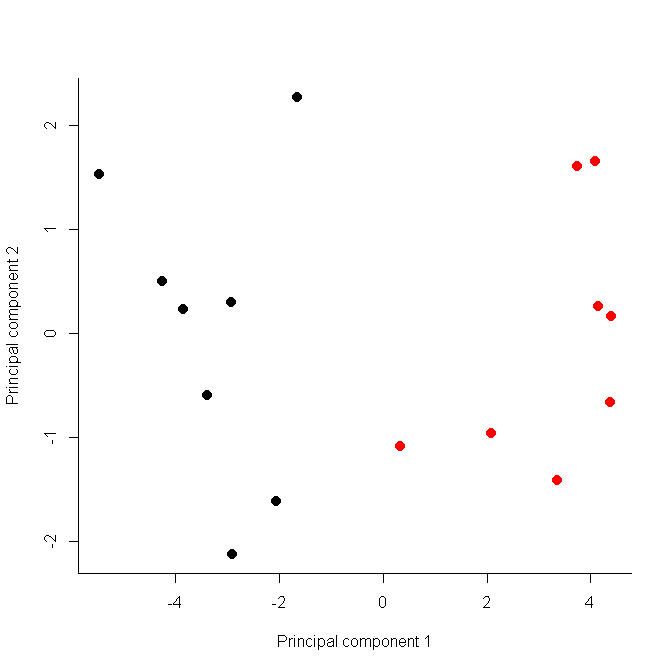

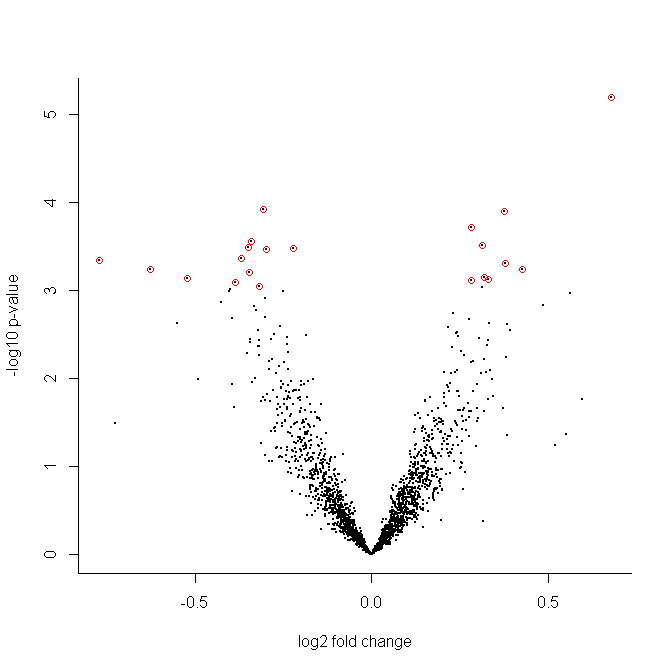

Supplement: Supplementary file 1 [file Data_Sheet_1.zip › Supplementary Material data sheet 1/Suppl fig 2c.docx]

**Hippocampus**

**Veh vs WT**


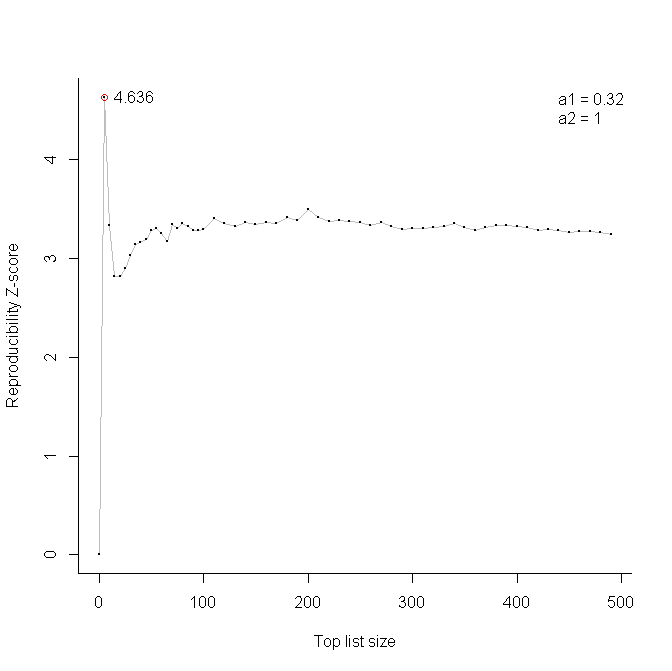


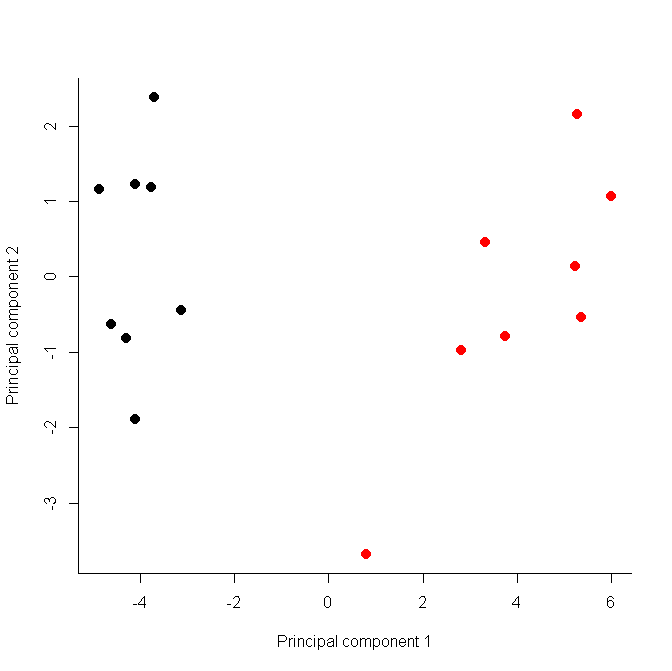


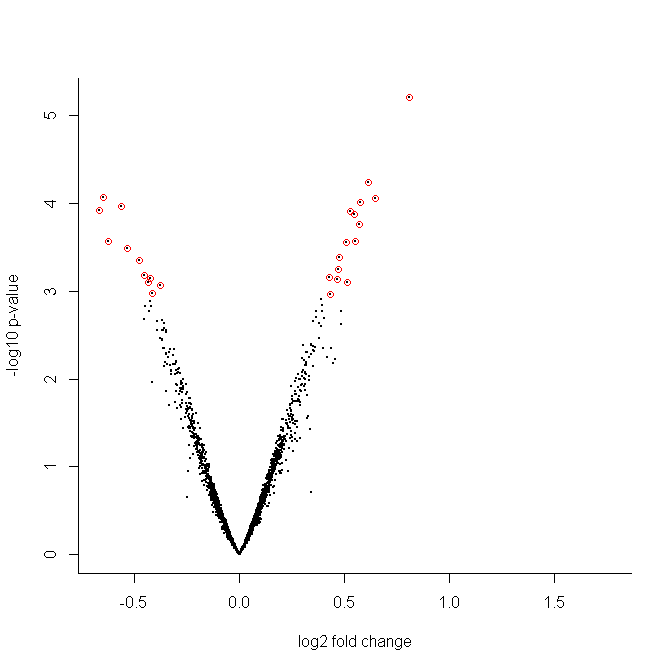


**Hippocampus**

**DCE vs VeH**


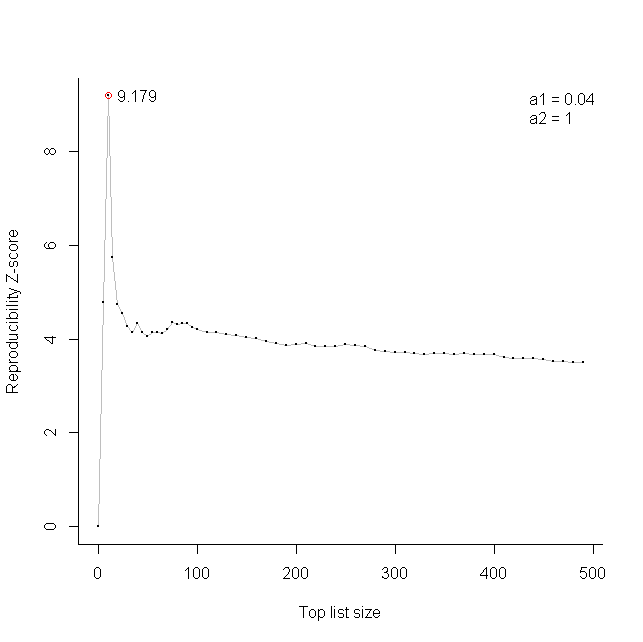


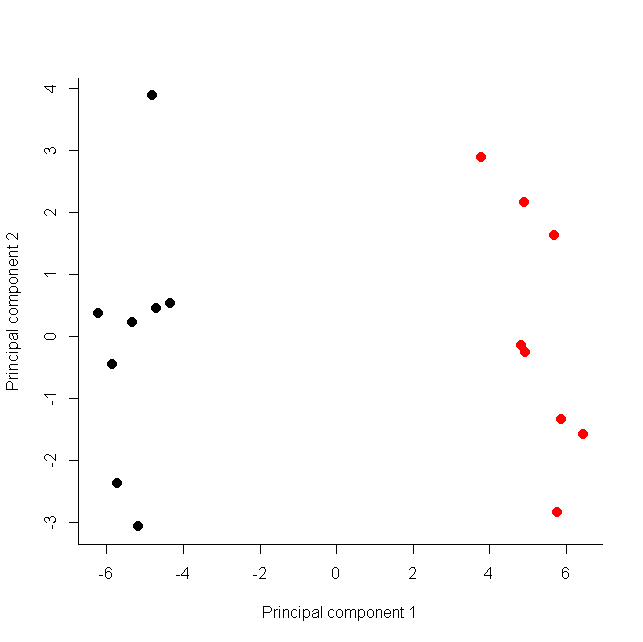


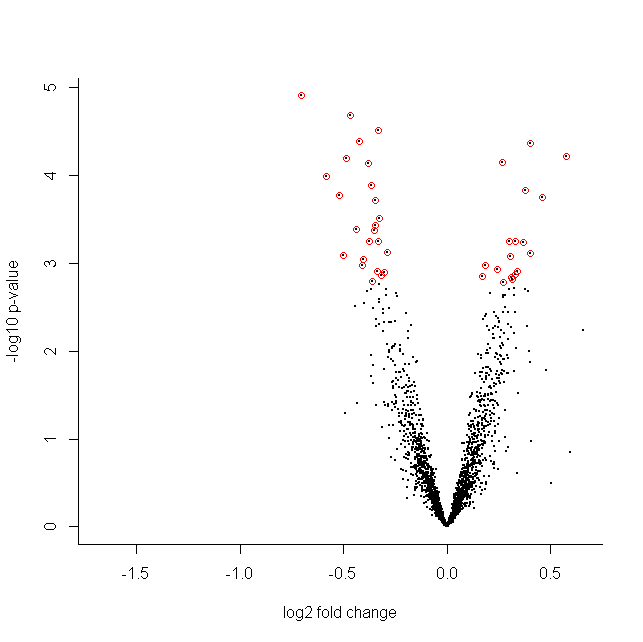

Supplement: Supplementary file 1 [file Data_Sheet_1.zip › Supplementary Material data sheet 1/Suppl fig 2d.docx]

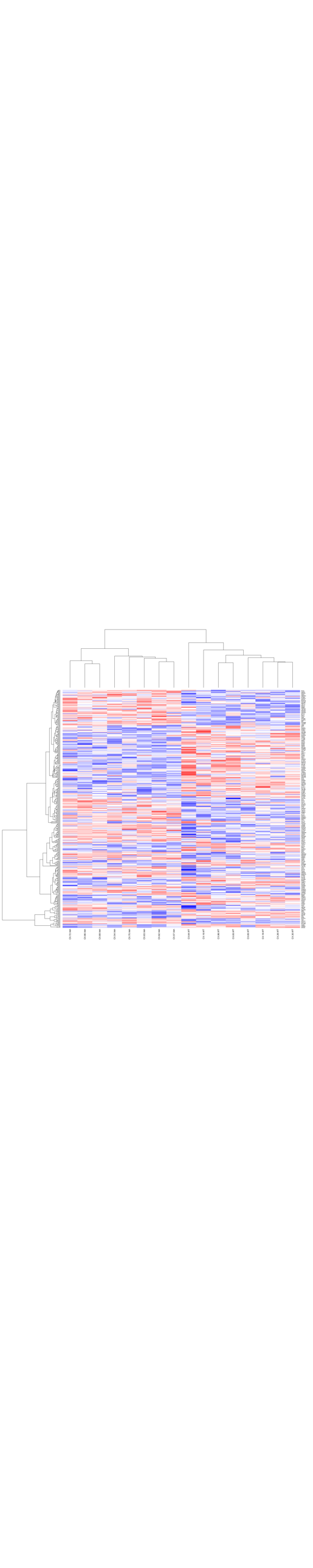

Supplement: Supplementary file 1 [file Data_Sheet_1.zip › Supplementary Material data sheet 1/Suppl Fig 3a.pdf]

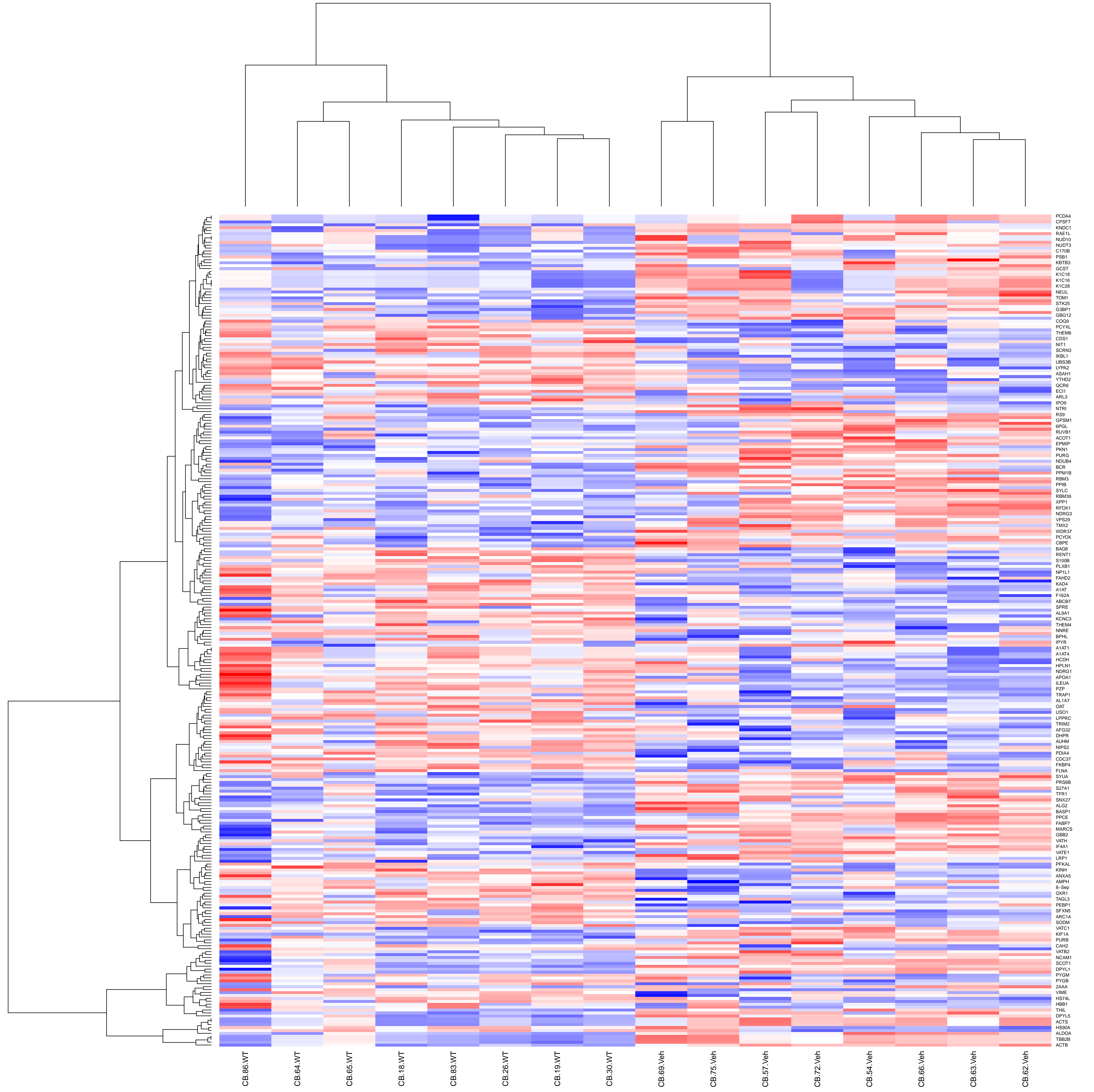

Supplement: Supplementary file 1 [file Data_Sheet_1.zip › Supplementary Material data sheet 1/Suppl Fig 3c.pdf]

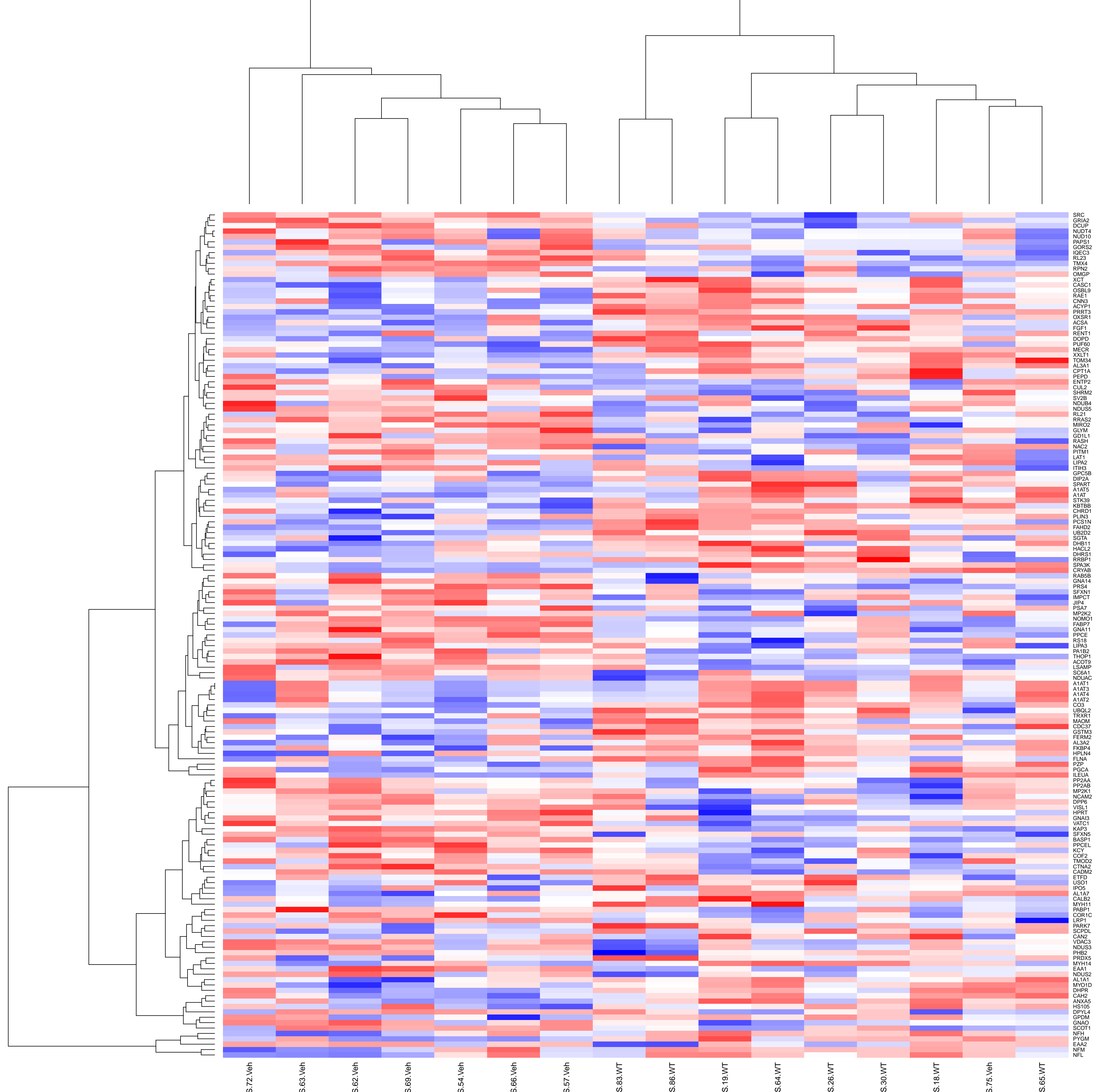

Supplement: Supplementary file 1 [file Data_Sheet_1.zip › Supplementary Material data sheet 1/Suppl Fig 3e.pdf]

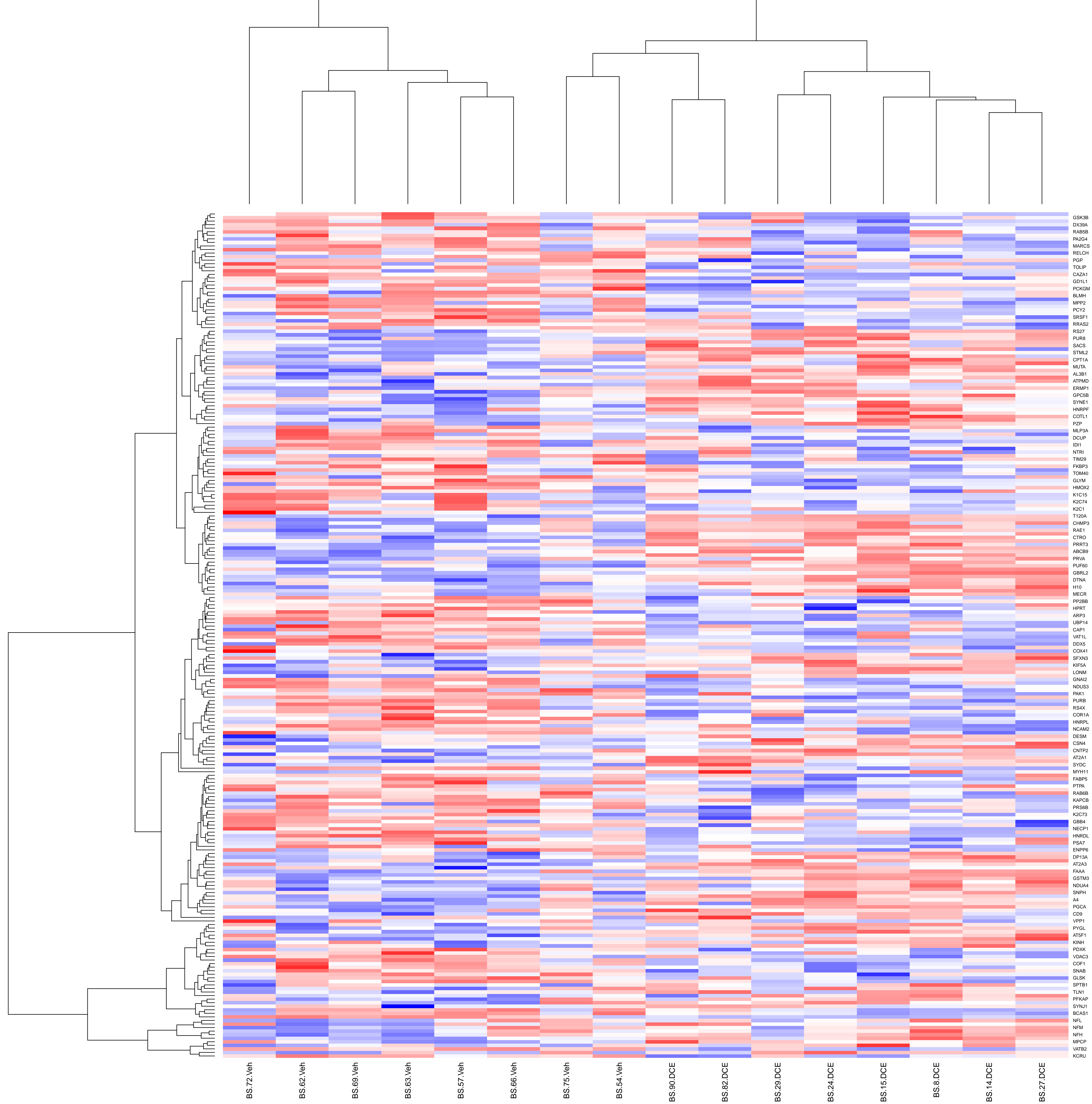

Supplement: Supplementary file 1 [file Data_Sheet_1.zip › Supplementary Material data sheet 1/Suppl Fig 3f.pdf]

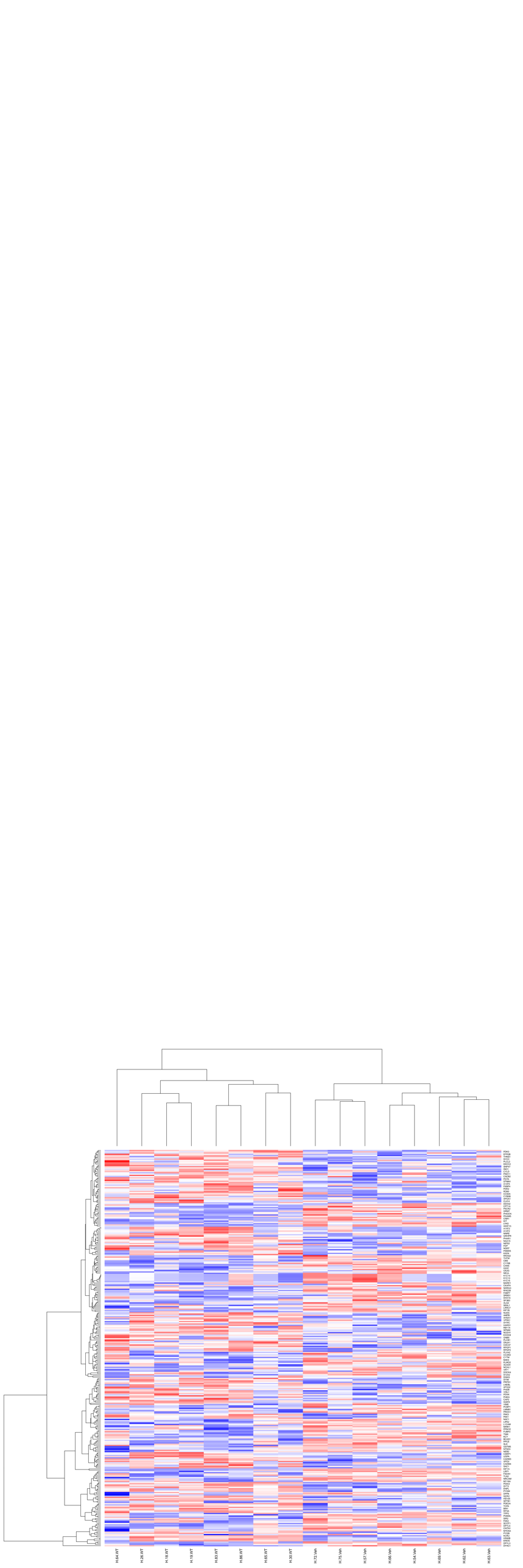

Supplement: Supplementary file 1 [file Data_Sheet_1.zip › Supplementary Material data sheet 1/Suppl Fig 3g.pdf]

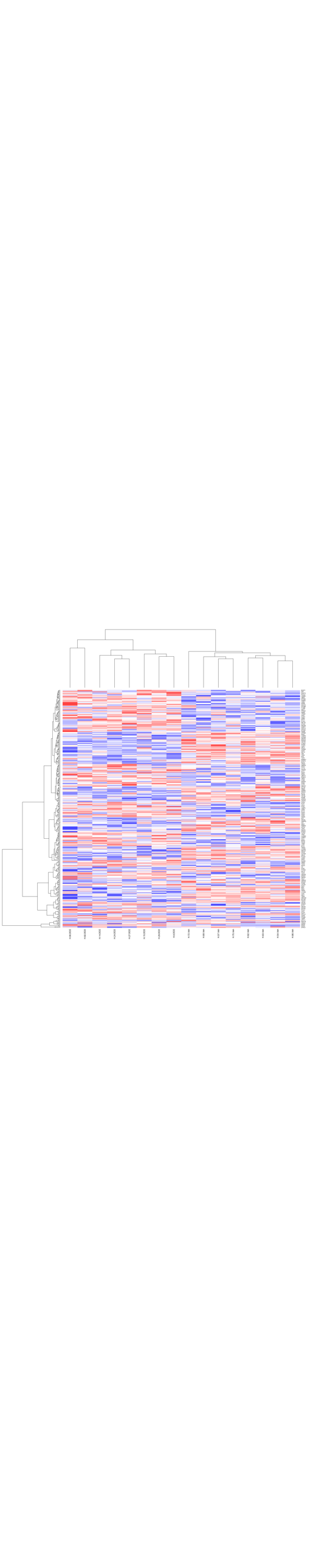

Supplement: Supplementary file 1 [file Data_Sheet_1.zip › Supplementary Material data sheet 1/Suppl Fig 3h.pdf]

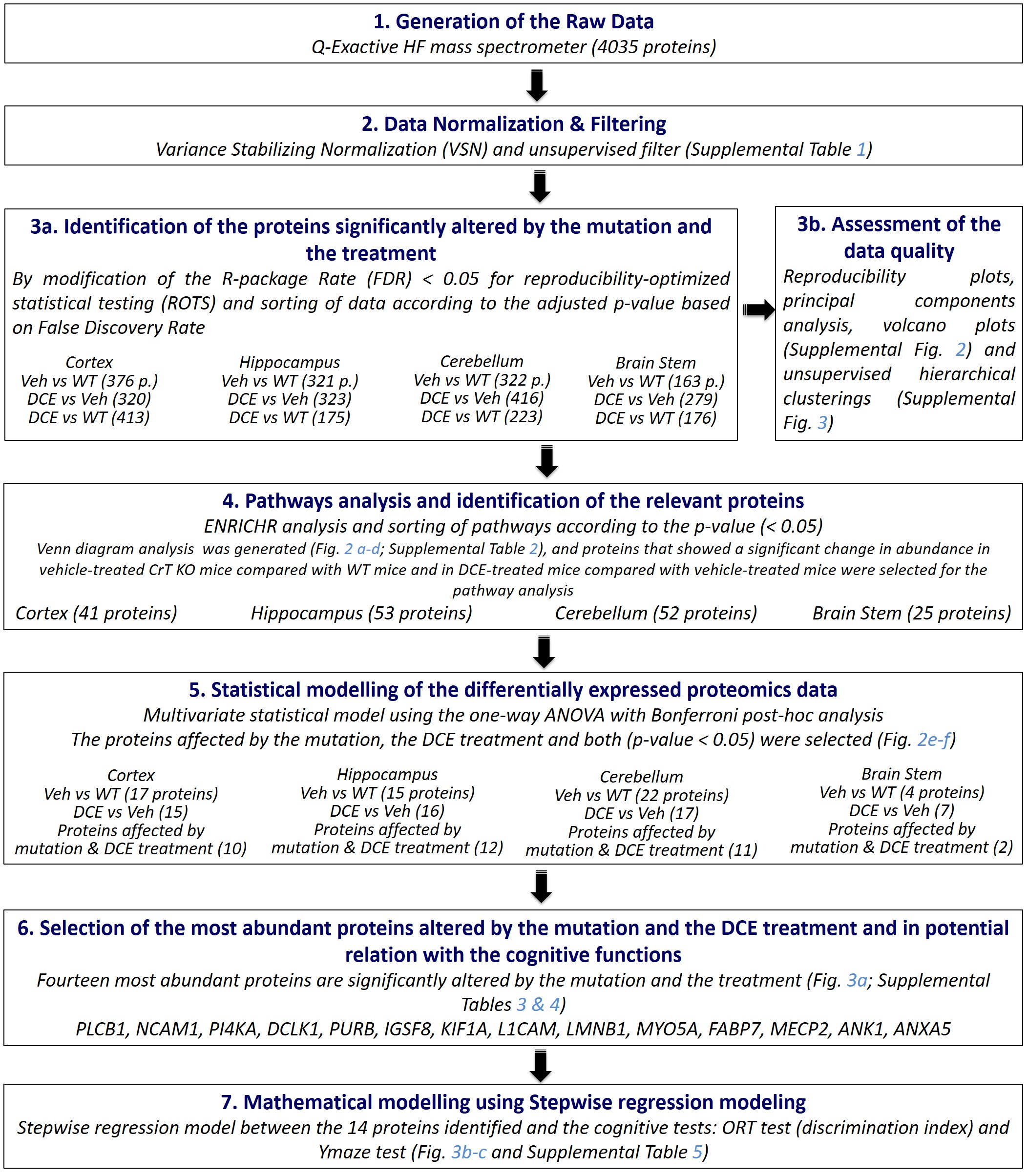

Supplement: Supplementary file 1 [file Data_Sheet_1.zip › Supplementary Material data sheet 1/Supplemental Fig 1.jpg]
